# IL-10 Beyond Immunosuppression: Unraveling Spatial, Cellular, and Metabolic Mechanisms of Gut Protection

**DOI:** 10.3390/ijms27219472

**Published:** 2026-10-23

**Authors:** Yuchen Zhang, Xiaohui Liu, Boya Wang, Haoyuan Yin, Xiang Tian, Jiatong Zhao, Shuzi Xin, Hongli Wang, Yushuo Ren, Xinyue Liu, Jingyu Liu, Yuhang Bian, Sijin Gu, Haoxuan Zhang, Tong Wei, Lei Gao, Xin Lu, Hongwei Shang, Jingdong Xu

**Affiliations:** 1 Department of Clinical Medicine, School of Basic Medical Sciences, Capital Medical University, Beijing 100069, China; cherryzyc123@163.com (Y.Z.); yhy3616@163.com (H.Y.); 18610793068@163.com (X.T.); zhaojt416@163.com (J.Z.); 13621023142@163.com (J.L.); 13269695655@163.com (Y.B.); 15959296639@163.com (S.G.); haoxuanzhang2020@outlook.com (H.Z.); 18210728127@163.com (T.W.); 2 Department of Physiology and Pathophysiology, School of Basic Medical Sciences, Capital Medical University, Beijing 100069, China; m13484363443@163.com (X.L.); xinshuzi@gmail.com (S.X.); 18845857683@163.com (H.W.); 3 Key Laboratory of Carcinogenesis and Translational Research (Ministry of Education/Beijing), Department of Gastrointestinal Oncology, Peking University Cancer Hospital & Institute, Beijing 100142, China; wbysonnig@163.com; 4 Department of Intelligent Medical Engineering, School of Biomedical Engineering, Capital Medical University, Beijing 100069, China; bmi5@ccmu.edu.cn; 5 Experimental Centre for Morphological Research Platform, School of Basic Medical Sciences, Capital Medical University, Beijing 100069, China; luxin@ccmu.edu.cn

**Keywords:** IL-10, intestinal homeostasis, gut microbiota, metabolic regulation, epithelial barrier, inflammatory bowel disease, precision immunotherapy

## Abstract

Intestinal homeostasis requires immune tolerance, barrier integrity, tissue repair, and microbiota mutualism. Interleukin-10 (IL-10) is essential, but not merely as an immunosuppressant. Although IL-10 signals via the IL-10R—JAK1/TYK2—STAT3 axis, its outputs are cell-type and context dependent. In macrophages, IL-10 restrains IL-23 production, inflammasome activation, IL-1β release, and metabolic reprogramming. In T cells, it limits pathogenic T helper 17 cell (Th17) responses while stabilizing regulatory cells and promoting metabolically driven IL-10 production. In the epithelium, IL-10 maintains barrier integrity by limiting apoptosis, endoplasmic reticulum (ER) stress, and mucus defects. IL-10 and the gut microbiota engage in reciprocal regulation: microbial metabolites and structural components enhance IL-10 production and receptor responsiveness, whereas IL-10 signaling preserves microbial diversity and ecological resilience. This may help to explain why systemic recombinant IL-10 failed in Crohn’s disease (CD)—increasing ligand abundance alone is unlikely to recapitulate endogenous localization or restore refractory target cells. Emerging strategies aim to tune IL-10 signaling through gut-selective delivery, engineered cytokines, conditionally activated prodrugs, regulatory cell approaches, and microbiome or metabolic interventions. Clinical translation requires biomarkers of IL-10R expression, STAT3 responsiveness, macrophage metabolic state, microbial signatures, and acquired IL-10 hyporesponsiveness. Despite the established role of IL-10 in intestinal homeostasis, systemic IL-10 therapy has failed in clinical trials, and there is a lack of effective strategies to restore IL-10 signaling in a spatially and cell-specific manner. The spatially organized, metabolically conditioned homeostatic IL-10 signaling—largely inferred from model systems and awaiting direct spatial validation in human intestine—offers a roadmap for more precise therapy in inflammatory bowel disease and related intestinal disorders.

## 1. Introduction

In this review, we propose that interleukin-10 (IL-10) should be understood not merely as an anti-inflammatory cytokine, but as a spatially organized, cell-type-specific, and metabolically conditioned homeostatic pathway that coordinates immune regulation, barrier maintenance, and host–microbiota mutualism. We emphasize that this spatial framework is currently supported primarily by model systems, particularly mouse studies, and has not yet been directly mapped in human intestine. This organizing principle explains why simple ligand supplementation has failed and guides next-generation therapeutic strategies. The unmet medical need is clear: despite the critical role of IL-10 in intestinal homeostasis, systemic IL-10 therapy has failed in clinical trials, and there is currently no approved therapy that effectively restores IL-10 signaling in a spatially and cell-specific manner. This review aims to address this gap by synthesizing recent advances in spatial organization, cellular specificity, metabolic regulation, and host–microbiota interactions, and by proposing a framework for next-generation IL-10-based therapies.

Maintaining tolerance to commensals and dietary antigens while preserving protective immunity is essential for intestinal homeostasis, and its disruption drives inflammatory bowel disease (IBD). Given the increasing global burden of IBD and notable non-response rates to current therapies [1], defining the regulatory circuits that sustain mucosal immune homeostasis is urgent.

IL-10 is a pivotal component of this regulatory network. It signals through interleukin-10 receptor 1 (IL-10R1)/interleukin-10 receptor 2 (IL-10R2) to activate Janus kinase 1 (JAK1)/tyrosine kinase 2 (TYK2)—signal transducer and activator of transcription 3 (STAT3) pathway, suppressing pro-inflammatory cytokine transcription and antigen-presenting programs [2]. Yet, human genetic studies have shown that inactivating *IL10/IL10R* mutations cause severe, early-onset, refractory colitis, indicating that IL-10 signaling is not merely a modifier of inflammation but an irreplaceable requirement for mucosal immune regulation [3,4].

IL-10 is now recognized as a context-dependent regulator that shapes macrophage and T cell metabolism [5,6], responds to microbial signals [7], and participates in epithelial repair, fibrosis, and tumor immunity [8,9,10], acting as an integrative homeostatic mediator rather than merely an anti-inflammatory factor.

This review synthesizes evidence that IL-10 protects intestinal health through cell-type-specific immune regulation, metabolic programming, host–microbiota crosstalk, and stage-dependent tissue responses. We summarize IL-10R signaling, cellular sources, target-cell specificity, acquired hyporesponsiveness, barrier functions, and context-dependent roles in IBD, infection, fibrosis, and colorectal cancer (CRC), and then evaluate therapeutic strategies moving from systemic supplementation toward spatially and functionally controlled signaling.

This is a narrative review. Literature was identified through searches of PubMed and Elsevier (ScienceDirect) using the terms ‘IL-10’ combined with ‘intestine’, ‘gut’, ‘epithelium’, ‘macrophage’, ‘microbiota’, ‘metabolism’, ‘barrier’, ‘Crohn’s disease’, ‘ulcerative colitis’, and ‘colorectal cancer’. We prioritized foundational and discovery papers cited by key reviews, traced newer primary studies from their reference lists, and favored work published within the last five years where available, while retaining classical and seminal studies necessary to establish mechanistic context. No formal systematic-search or risk-of-bias appraisal was performed; accordingly, the synthesis reflects the authors’ interpretive framework rather than a comprehensive meta-narrative.

## 2. Core Principles of IL-10 Signaling

To understand the limited efficacy of systemic IL-10 supplementation, we must first define the cellular and molecular signaling architecture that allows IL-10 to function as a local, cell-specific regulator.

### 2.1. Cellular Sources and Spatial Organization

**Figure 1:**
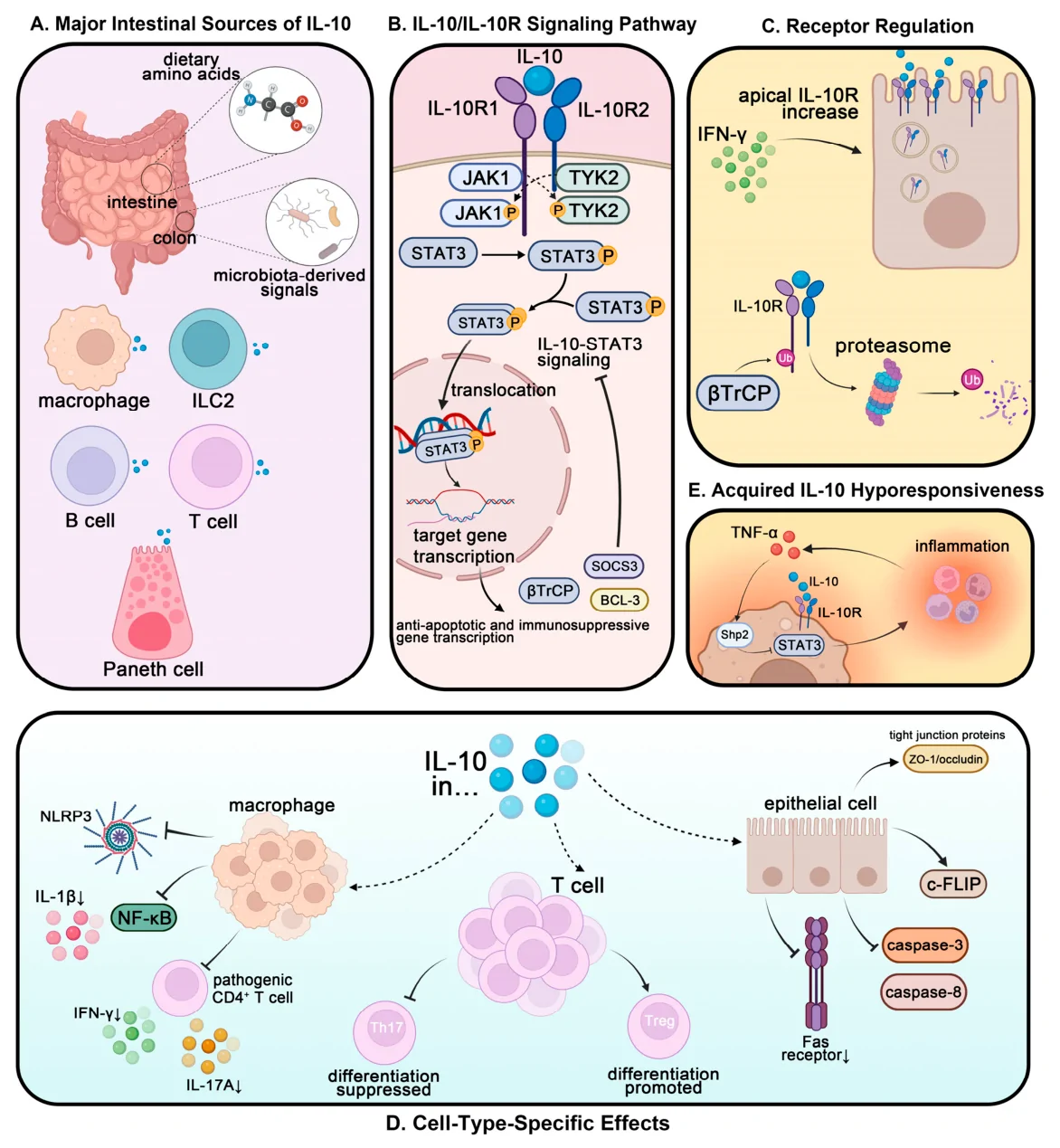
Core architecture and functional specialization of intestinal IL-10 signaling. (**A**) Proposed model of anatomical IL-10 sources in the intestine, based primarily on mouse studies; intestinal IL-10 is produced by multiple mucosal cell types, controlled by dietary amino acids in the small intestine and microbial signals in the colon. This spatial organization remains to be directly validated in human intestine. (**B**) IL-10 activates the IL-10R1/IL-10R2—JAK1/TYK2—STAT3 pathway for anti-inflammatory effects. (**C**) Responsiveness is regulated by IL-10R1 induction and degradation. (**D**) IL-10 protects the intestine via effects on macrophages, T cells, and epithelial barrier. IL-10 signaling in macrophages suppresses NLRP3 inflammasome and IL-1β production, thereby reducing IFN-γ and IL-17A production by pathogenic CD4^+^ T cells; in T cells, it suppresses Th17 differentiation and promotes Treg differentiation; in epithelial cells, it inhibits apoptosis and upregulates tight junction proteins. (**E**) Hyporesponsiveness and inflammation circuit. IL-10, interleukin-10; IL-10R1, interleukin-10 receptor 1; IL-10R2, interleukin-10 receptor 2; JAK1, Janus kinase 1; TYK2, tyrosine kinase 2; STAT3, signal transducer and activator of transcription 3; SOCS3, suppressor of cytokine signaling 3; βTrCP, beta-transducin repeat-containing protein; BCL-3, B cell lymphoma 3 protein; IFN-γ, interferon gamma; Ub, ubiquitin; NLRP3, NOD-like receptor protein 3; Th17, T helper 17 cells; Treg, regulatory T cells; c-FLIP, cellular FLICE-like inhibitory protein; caspase-8, cysteine-aspartic acid protease-8; caspase-3, cysteine-aspartic acid protease-3; ZO-1, zonula occludens-1.

Building on its established role in immune regulation, IL-10 is produced by diverse innate and adaptive immune cells in the gut [2] (Figure 1A), with production and responsiveness organized by tissue location, cellular context, and functional demand.

Myeloid cells are a major compartment. Colonic macrophages require IL-10R signaling for regulatory function. Though macrophage-derived IL-10 is mainly dispensable for general systemic homeostasis, loss of IL-10R signaling in CX3CR1^+^ macrophages triggers severe spontaneous colitis (mouse, in vivo and ex vivo) [11]. Nevertheless, IL-10-producing macrophages also contribute to barrier maintenance in specific contexts. Early after small-intestinal injury, MHCII^+^CD64^+^Ly6C^low^ macrophages serve as a principal source of IL-10, and their production of IL-10 promotes restoration of epithelial integrity (mouse, in vivo and ex vivo) [12]. IL-10 signaling limits local memory T cell (Tmem) reactivation in small-intestinal CD11c^+^ myeloid cells, and protects against colonic commensal-driven colitis (mouse, in vivo and ex vivo) [13]. These observations indicate that the same receptor pathway can support distinct protective functions in different intestinal segments.

Beyond macrophages, the gut harbors additional, physiologically important sources of IL-10. Intestinal group 2 innate lymphoid cells (ILC2s) provide an inducible source of IL-10 that can be further enhanced by cytokines such as IL-2, IL-4, and IL-27, as well as the neuropeptide neuromedin U (NMU) (mouse, in vivo and in vitro) [14]. In mouse small-intestinal organoids, a subpopulation of Paneth cells can produce IL-10 and a possible autocrine IL-10R1-dependent circuit has been proposed that may sustain antimicrobial protein expression (mouse organoids) [15]; this remains to be established in vivo and in human intestine. Among adaptive immune cells, IL-10-producing B cells can be activated by resident enteric microbiota through Toll-like receptor 2 (TLR2)/MyD88/phosphoinositide 3-kinase (PI3K) signaling (mouse, in vivo and in vitro) [16]. T cell-derived IL-10 is particularly important for restraining T cell responses, whereas non-T cell-derived IL-10 plays distinct roles in innate inflammatory settings such as endotoxin shock and skin irritation (mouse, in vivo and ex vivo) [17].

Synthesizing separate mouse studies, a proposed model posits segment-specific anatomical logic for IL-10 sources (Figure 1A): small-intestinal IL-10-producing macrophages can be modulated by dietary amino acids (mouse, in vivo and ex vivo) [18], whereas colonic ones depend on commensal microbiota (mouse, in vivo and ex vivo) [19]. This organization is inferred from non-spatial, largely mouse, studies and remains to be confirmed by direct spatial mapping in human intestine, where such evidence is currently limited. Moreover, microbiota-specific T helper cells 17 (Th17) have been shown to acquire an IL-10^+^ regulatory phenotype via c-MAF-dependent and local IL-10 signaling in the intestinal lamina propria (mouse, in vivo and in vitro) [20], suggesting additional, potentially segment-specific regulatory layers that similarly await direct spatial validation in human tissues.

### 2.2. Receptor Dynamics: Assembly, Turnover, and Contextual Licensing

Having mapped IL-10’s cellular sources, we now examine the receptor mechanisms by which IL-10 exerts its local effects. IL-10 signals through a heterotetrameric receptor (two IL-10R1 and two IL-10R2 chains), activating JAK1/TYK2 and downstream STAT3 phosphorylation, dimerization, and nuclear translocation [2,21,22,23] (Figure 1B). IL-10 responses vary by cell type due to differences in receptor abundance, chain usage, and signaling intensity (human, in vitro) [24], providing a mechanistic basis for the cell-type-specific effects of IL-10. For instance, the local inflammatory environment dynamically regulates IL-10R ability. In polarized intestinal epithelial cells, interferon gamma (IFN-γ) selectively induces apical IL-10R1 expression (human cell lines), enabling epithelial responses to luminal or mucosal IL-10 signals, decreasing intestinal permeability and attenuating DSS-induced colitis (mouse, in vivo) [25] (Figure 1C).

Additionally, IL-10 signaling is actively terminated. The length of downstream signaling is limited by IL-10R1’s quick internalization and proteasome-dependent destruction upon ligand interaction (cell lines) [26]. This process involves recruitment of a beta-transducin repeat-containing protein (βTrCP)-containing E3 ubiquitin ligase to IL-10R1, which facilitates receptor ubiquitination and instability (cell lines) [27] (Figure 1C).

Thus, cell-type-specific signaling, receptor turnover, local inflammatory signals, and receptor availability all influence IL-10 responsiveness. Defects in receptor induction, degradation, or downstream signaling may underlie inadequate responses.

### 2.3. Cell-Type-Specific Responses

Receptor availability and turnover define the signaling context, but the response elicited by IL-10 depends on the recipient cell type (Figure 1D).

Genome-wide analyses show IL-10’s anti-inflammatory program varies across myeloid subsets. In macrophages, IL-10 predominantly suppresses nuclear factor κB (NF-κB)-dependent inflammatory genes; in dendritic cells and mast cells, it indirectly interferes with transcriptional programs; and in neutrophils, both regulatory modes appear to operate in parallel (mouse, in vitro and ex vivo) [28].

These observations reflect distinct genetic models and intestinal compartments rather than a contradiction. Global or myeloid-wide loss of macrophage-derived IL-10 is largely dispensable for baseline homeostasis, whereas the requirement for IL-10R signaling in specific myeloid subsets is strictly conditional [11]. Separately, in the steady-state colon, macrophages engage an autocrine IL-10 loop to restrain IL-23 (mouse, ex vivo) [29] and maintain commensal tolerance (mouse, in vivo and ex vivo) [19]. The requirement is therefore context-, location-, and model-dependent: autocrine IL-10 is critical in the colonic homeostatic/low-grade inflammatory setting but is not a universal prerequisite for all macrophage functions, and conclusions should not be generalized across intestinal segments or inflammatory states.

This autocrine circuit operates through multiple regulatory layers. Macrophage regulation of inflammasome activity becomes compromised in the absence of IL-10 signaling. IL-10R-deficient macrophages show enhanced NOD-like receptor protein 3 (NLRP3) inflammasome activity, causing caspase-8-dependent IL-1β release [30], which drives pathogenic CD4^+^ T cells to produce IFN-γ and IL-17A [30]. IL-10 also restricts post-transcriptional and post-translational inflammatory checkpoints. Once this control is lost, the IL-1β–T cell axis self-amplifies.

IL-10 functions differently in the adaptive immune compartment. In T cells, its anti-inflammatory effects are likewise subset dependent. IL-10R signaling directly suppresses Th17 differentiation (mouse, in vivo and in vitro) [31], while promoting regulatory T cell (Treg) differentiation (human cells) [32], thereby limiting pathogenic Th17 responses (mouse, in vivo) [33]. However, IL-10R signaling is not strictly required for Treg generation in vitro (human) [34]. Furthermore, IL-10R-deficient Tregs become unstable and acquire IL-17A-producing features in the presence of IL-1β (human cells) [34], indicating IL-10 maintains Treg lineage stability in inflamed tissues.

Epithelial cells can interpret IL-10 as a survival signal. IL-10 downregulates Fas receptor expression, increases cellular FLICE-like inhibitory protein (c-FLIP) expression, and suppresses caspase-8 and caspase-3 activation, thereby interrupting the extrinsic apoptosis (mouse cell line) [35]. In addition to limiting apoptosis, IL-10 upregulates tight-junction proteins to epithelial barrier maintenance [36].

### 2.4. Acquired IL-10 Hyporesponsiveness: When Cells Stop Answering

However, even when IL-10 is abundant and derived from an appropriate cellular source, target cells may fail to transduce IL-10 signals. In inflamed tissues, target cells can become hyporesponsive to IL-10—a state of impaired IL-10 responsiveness that shifts the therapeutic problem from ligand quantity to cellular responsiveness. We use the term ‘acquired IL-10 hyporesponsiveness’ rather than ‘acquired IL-10 resistance’ to reflect the fact that this state has not been fully established in vivo in humans.

One molecular mediator of this acquired IL-10 hyporesponsiveness is the phosphatase SH2 domain-containing protein tyrosine phosphatase 2 (Shp2). In colonic macrophages from patients with IBD, Shp2 expression is upregulated and disrupts canonical IL-10—STAT3 signaling, thereby reducing macrophage responsiveness to IL-10. Notably, tumor necrosis factor alpha (TNF-α) can induce Shp2 expression (human cells) [37], establishing a feedforward inflammatory loop in which pro-inflammatory cytokines promote Shp2 upregulation, Shp2 attenuates IL-10 responsiveness, and impaired IL-10 signaling further amplifies intestinal inflammation (Figure 1E).

Microbial stimulation also induces IL-10 hyporesponsiveness in monocytes via an IL-1/IL-10 network that controls IL-23 output; acquired IL-10 hyporesponsiveness disrupts this balance and allows IL-23 production (human cells) [38], demonstrating that genetically intact immune cells can become functionally refractory in response to environmental and microbial cues.

Clinical observations reinforce the relevance of IL-10 hyporesponsiveness in human intestinal inflammation. In active ulcerative colitis (UC), both IL-10 mRNA expression and the frequency of IL-10^+^ mucosal T cells are elevated, yet inflammation persists rather than resolves (human cells) [39].

Counterintuitively, high pre-treatment mucosal IL-10 expression has been associated with poor response to biologic therapy in IBD (human cells) [40], this may reflect a general inflamed tissue state with reduced IL-10 responsiveness rather than proven receptor incompetence. Functional evidence from IL-2-deficient mice further supports this concept: microbial antigens induce robust IL-10 production in the colon, but the cytokine fails to suppress IFN-γ production (mouse, ex vivo) [41]. Acquired IL-10 hyporesponsiveness provides a mechanistic explanation for the limited efficacy of first-generation replacement strategies. Once target cells in inflamed tissue become refractory to IL-10, merely increasing cytokine levels alone cannot restore immune regulation; restoring cellular responsiveness becomes equally critical as shown in Figure 1 and the cell-type-specific responses to IL-10 are summarized in Table 1.

## 3. Metabolic and Barrier Functions of IL-10

Beyond signaling and hyporesponsiveness, metabolism determines whether target cells can execute the resulting regulatory program. IL-10 reshapes metabolic and stress-response programs in immune and epithelial cells, thereby defining a metabolic state permissive for durable immune restraint, epithelial survival, and tissue repair [49,50,51].

### 3.1. Macrophage Metabolic Tolerance: Lipids, Mitochondria, and Epigenetic Imprinting

Macrophages are a central target of IL-10-mediated metabolic regulation. IL-10 serves as a checkpoint for macrophage lipid metabolism, preserving fatty acid desaturation and preventing the pathological accumulation of inflammatory sphingolipid. In IL-10-deficient macrophages, impaired de novo synthesis of monounsaturated fatty acid (MUFA) leads to the accumulation of saturated very-long-chain ceramides, which sustain Rel proto-oncogene, NF-κB subunit (REL)-dependent inflammatory gene expression. Conversely, MUFA supplementation restores lipid metabolic balance and limits inflammation (mouse, in vivo and vitro) [5], extending IL-10’s role beyond transcriptional suppression to the maintenance of lipid metabolic homeostasis.

This metabolic function also explains the persistent inflammatory imprint in IL-10-deficient intestinal macrophages. IL-10 deficiency induces stable chromatin alterations that cannot be fully reversed by subsequent exogenous IL-10 (mouse, ex vivo) [52]. Although the precise links between sphingolipid dysregulation and chromatin remodeling remain to be established, IL-10 may prevent the establishment of a durable metabolic-epigenetic inflammatory state.

**Figure 2:**
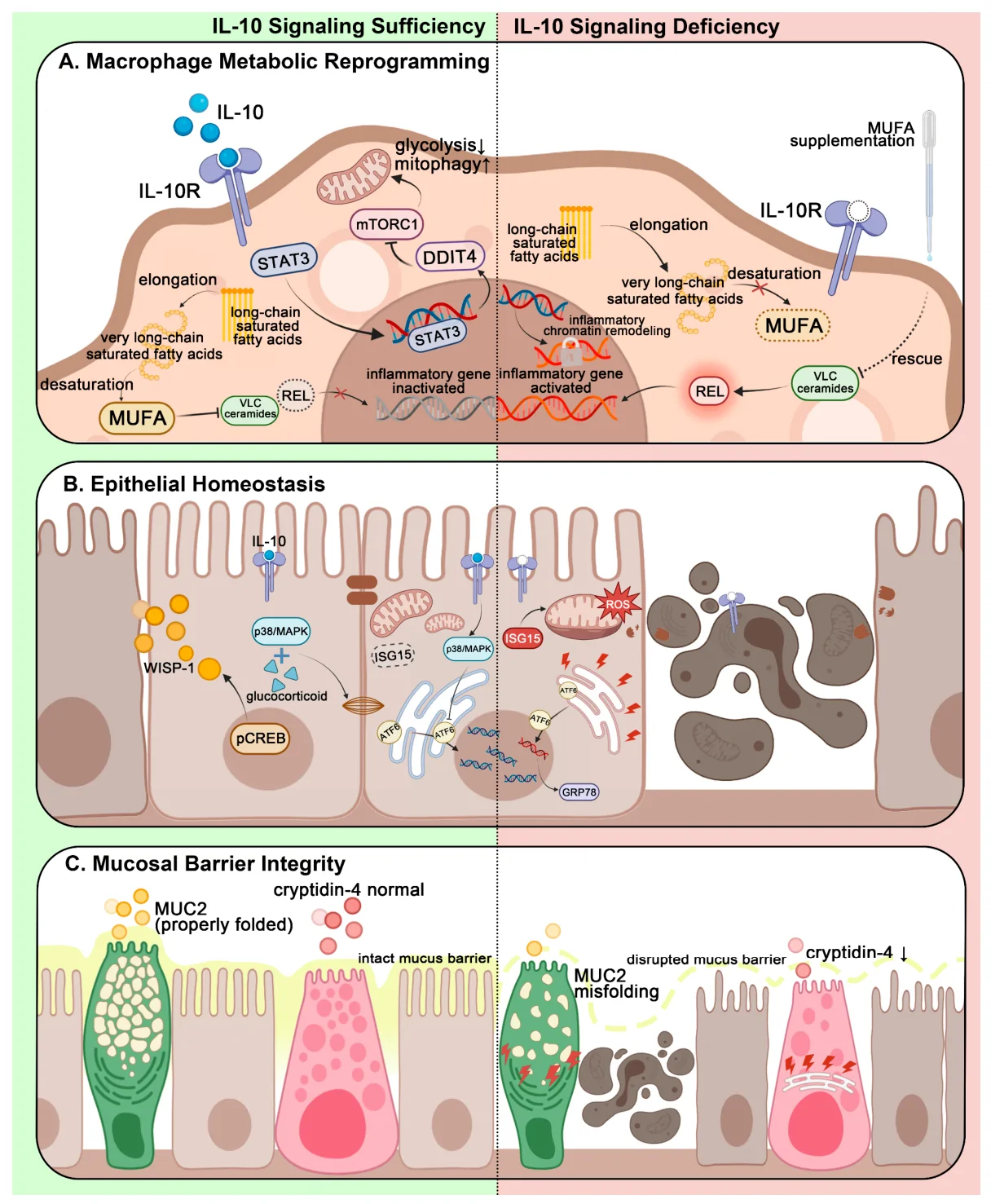
IL-10 protects the intestine via distinct immune and epithelial mechanisms. (**A**) In macrophages, IL-10 limits mTORC1-glycolytic activation and VLC ceramide-driven inflammation, while promoting mitophagy and MUFA synthesis. (**B**) In epithelial cells, IL-10 promotes repair and stress adaptation via phosphorylated CREB and p38/MAPK signaling, while suppressing ER stress. (**C**) At the mucosal surface, IL-10 maintains goblet and Paneth cell function, ensuring MUC2 folding, mucus integrity, and barrier defense. IL-10, interleukin-10; IL-10R, interleukin-10 receptor; STAT3, signal transducer and activator of transcription 3; mTORC1, mechanistic target of rapamycin complex 1; DDIT4, DNA damage-inducible transcript 4; VLC ceramides, very-long-chain ceramides; MUFA, monounsaturated fatty acid; REL, Rel proto-oncogene, NF-κB subunit; WISP-1, WNT1-inducible signaling pathway protein 1; CREB, cAMP response element-binding protein; ER, endoplasmic reticulum; ROS, reactive oxygen species; ISG15, interferon-stimulated gene 15; GRP78, glucose-regulated protein 78; ATF6, activating transcription factor 6; MUC2, mucin 2.

IL-10 further reprograms macrophage inflammatory metabolism by inhibiting lipopolysaccharide (LPS)-induced glycolytic reprogramming and suppressing mechanistic target of rapamycin complex 1 (mTORC1) activity through DNA damage-inducible transcript 4 (DDIT4), promoting mitophagy and reducing reactive oxygen species (ROS) accumulation and inflammasome activation (mouse and human cells) [42] (Figure 2A). The relationship between IL-10 and metabolism is bidirectional. Mitochondrial complex III-derived superoxide is required for optimal IL-10 secretion in a cell-intrinsic manner in macrophages (mouse, in vitro) [53], and activation of pyruvate kinase M2 (PKM2) enhances macrophage IL-10 production through glycolytic ATP generation and adenosine-A2a receptor signaling (mouse, in vitro) [54]. These effects appear to be primarily cell-intrinsic, although indirect contributions from other cell types cannot be excluded.

This metabolic dependence has direct implications for therapeutic macrophage reprogramming. Anti-TNF therapy increases macrophage IL-10 expression via an Fc gamma receptor (FcγR)-dependent mechanism and promotes a CD206^+^ regulatory phenotype, with efficacy dependent on intact macrophage IL-10 signaling (mouse and human cells) [55]. Failure of this axis may limit homeostasis restoration.

### 3.2. Epithelial Resilience: From ER Stress to Wound Closure

The intestinal epithelium faces continuous microbial, dietary, mechanical, and inflammatory stress. Consistent with the critical role of IL-10 in epithelial protection, IL-10-deficient mice develop chronic enterocolitis with mucosal hyperplasia and aberrant epithelial MHC class II expression (mouse, in vivo) [56] (Figure 2B).

Mechanically, epithelial effects of IL-10 are best separated into two categories: cell-intrinsic, direct IL-10R signaling and indirect, inflammation-secondary protection. (i) Cell-intrinsic actions: epithelial cells express IL-10R1 (inducibly on the apical surface under IFN-γ, as discussed in Section 2.2) and respond directly—limiting ER stress by blocking activating transcription factor 6 (ATF6) [45], suppressing the Fas/caspase-8 apoptosis pathway (detailed in Section 2.3), and upregulating tight-junction proteins including zonula occludens-1 (ZO-1) and occludin [36]. (ii) Indirect, inflammation-secondary actions: by suppressing mucosal myeloid and T cell inflammation, IL-10 lowers the cytokine burden (TNF-α, IFN-γ) that drives epithelial apoptosis and barrier loss (human and non-human primate colon explants) [57,58]. Both modes operate in vivo and should not be conflated when interpreting epithelial protection.

#### 3.2.1. ER and Mitochondria: Quality Control Under Stress

IL-10 protects epithelial cells by restraining endoplasmic reticulum (ER) stress. Inflammatory TNF activates the ER stress sensor ATF6, promoting its nuclear translocation and binding to the glucose-regulated protein 78 (GRP78) promoter. IL-10 blocks nuclear translocation of ATF6 via p38/STAT3 signaling, thereby reducing ER stress in intestinal epithelial cells (mouse, in vitro) [45]. This effect is particularly critical in secretory epithelial lineages. Blocking IL-10 signaling rapidly induces mucin 2 (MUC2) misfolding and compromises the mucus barrier (human cell lines) [46].

IL-10 also promotes mitochondrial quality control. Recent evidence suggests that IL-10 reduces interferon-stimulated gene 15 (ISG15) expression and enhances autophagy, improving mitochondrial function and limiting oxidative stress and epithelial apoptosis in UC (human cell lines) [59]. Conversely, autophagy can regulate IL-10R signaling. Autophagy-related 16-like 1 (ATG16L1) interacts with the IL-10R2 subunit through its WD40 domain, modulating IL-10R trafficking and signal strength (human and mouse cell lines) [60,61]. These insights place IL-10 within a reciprocal network linking ER stress, mitochondrial integrity, autophagy, and epithelial survival.

#### 3.2.2. Wound Repair: From Immune Sensing to Epithelial Closure

Beyond preventing stress-induced damage, IL-10 also contributes to epithelial repair after mucosal injury. Damage-responsive resident macrophages produce IL-10, which activates epithelial cAMP response element-binding protein (CREB) and induces WNT1-inducible signaling pathway protein 1 (WISP-1) production, promoting epithelial proliferation and wound closure (human and mouse cell lines, human enteroids) [8], establishing a direct macrophage–epithelial axis that converts immune sensing into tissue repair.

IL-10 restores tight junction proteins like ZO-1, occludin, and E-cadherin (mouse, in vivo and ex vivo) [36], cooperates with corticosteroids via p38/MAPK signaling (human cell lines) [62], and supports MUC2 synthesis and Paneth cell function (mouse, in vivo) [47,48], maintaining epithelial barrier competence prior to the inflammatory injury (Figure 2C).

### 3.3. T Cell Regulation: Metabolic Fuels for Lineage Stability

In T cells, IL-10 regulates effector cytokine production while supporting lineage stability and metabolic fitness. IL-10 induces Blimp-1 in Th2 cells via STAT3, forming an autoregulatory loop that enhances IL-10 production while limiting excessive T cell expansion by antagonizing STAT5-dependent programs (mouse, in vivo and in vitro) [63]. IL-10 also directly restrains Th17 cells, and both Foxp3^−^ type 1 regulatory T (Tr1) cells and Foxp3^+^ Tregs suppress pathogenic Th17 and Th1-like Th17 cells in an IL-10-dependent manner (mouse, in vivo and in vitro) [31].

T cell metabolism further influences IL-10 production. Dichloroacetate (DCA) increases IL-10 production by CD4^+^ T cells, accompanied by enhanced maximal oxygen consumption and reduced lactate secretion. Mechanistically, this response involves increased aryl hydrocarbon receptor (AHR) expression and reduced expression of the transcriptional repressor Bhlhe40; however, AHR inhibition does not abolish DCA-induced IL-10 upregulation, indicating that multiple metabolic pathways can converge on IL-10 transcription (mouse, in vivo and in vitro) [6]. Thus, IL-10 regulation in T cells is intimately linked to metabolic state.

### 3.4. Fibroblasts and Innate Lymphoid Cells: Emerging Metabolic Targets of IL-10

Beyond macrophages, epithelial cells, and T cells, IL-10 also regulates intestinal fibroblasts and ILCs, although the metabolic basis of these effects remains less well characterized.

Intestinal fibroblasts are key drivers of fibrosis, and fibroblast-specific deletion of *IL10R1* increases Type I and Type II IFN signaling in colonic fibroblasts, with aged mice developing spontaneous chronic colitis and subsequent fibrosis, suggesting that IL-10/IL-10R1 axis limits large intestinal pathology at least in part by suppressing IFN signaling in fibroblasts (mouse, in vivo and ex vivo) [64]. However, this study does not establish a direct metabolic mechanism. In skin, IL-10-treated fibroblast-derived apoptotic vesicles enhance mitochondrial oxidative phosphorylation (OXPHOS) and suppress glycolysis in fibroblasts (rat, in vivo and in vitro) [65]. Whether similar metabolic reprogramming occurs in intestinal fibroblasts remains to be tested.

Rorc-expressing ILCs, including ILC3s, produce IL-10 during intestinal inflammation and contribute to immune homeostasis. Mice with IL-10 deficiency specifically in Rorc-expressing ILCs exhibit altered intestinal macrophages and exacerbated experimental colitis, and adoptive transfer of IL-10-sufficient ILC3s rescued both the macrophage phenotype and disease severity (mouse, in vivo and ex vivo) [66]. These findings establish that ILC3-derived IL-10 restricts intestinal inflammation. The production of IL-10 by ILCs is metabolically regulated. In lung ILC2s, IL-10 production requires a shift from fatty acid oxidation to glycolysis and depends on Blimp-1 and cMaf (mouse, in vivo and in vitro) [67]. Calcitriol reprograms bone marrow ILC progenitors via a VDR–SYK–PKM2 axis to generate intestinal IL-10^+^IL-22^+^ ILC3s, with PKM2 linking metabolic reprogramming to *IL10* transcription and OXPHOS (mouse, in vivo and in vitro) [68]. However, direct evidence for IL-10-mediated metabolic reprogramming of human intestinal fibroblasts and ILC3s is still limited, and further studies are needed to define the molecular pathways involved.

### 3.5. Intercellular Metabolic Crosstalk in IL-10-Mediated Protection

The metabolic effects of IL-10 on macrophages, epithelial cells, and T cells are not isolated but are interconnected through soluble mediators and metabolic intermediates. For example, IL-10-mediated metabolic reprogramming in macrophages—such as promotion of mitophagy and maintenance of lipid homeostasis—can indirectly affect epithelial cells by reducing the release of proinflammatory cytokines (e.g., IL-1β, TNF-α) and reactive oxygen species that drive epithelial apoptosis and barrier loss [5,42]. In addition, macrophage-derived IL-10 promotes epithelial WISP-1 production, which supports epithelial proliferation and wound closure [8], indicating a direct macrophage–epithelial axis that converts immune metabolic sensing into tissue repair.

Conversely, IL-10-mediated metabolic regulation in T cells can influence macrophage activation. IL-10 suppresses Th17 differentiation and promotes Treg stability [31,32], thereby reducing the production of proinflammatory cytokines (e.g., IL-17A, IFN-γ) that activate macrophages. Metabolic programs in T cells—such as DCA-driven oxidative metabolism and AHR/Bhlhe40-dependent IL-10 production [6]—can reinforce regulatory circuits that limit macrophage-driven inflammation. These bidirectional interactions suggest that IL-10 acts as a metabolic coordinator across multiple cell types, rather than exerting isolated effects on individual populations.

However, the precise molecular mediators of this crosstalk remain incompletely defined, and future studies using co-culture systems and spatial metabolomics are needed to fully elucidate these interactions.

## 4. The Dialog Between IL-10 and the Gut Microbiota

Gut microbiota and mucosal immunity engage in reciprocal regulation. IL-10 mediates bidirectional host–microbiota symbiosis, maintaining microbial diversity and ecological stability.

### 4.1. Microbial Inputs: Metabolites and Structural Signals That Boost IL-10

**Figure 3:**
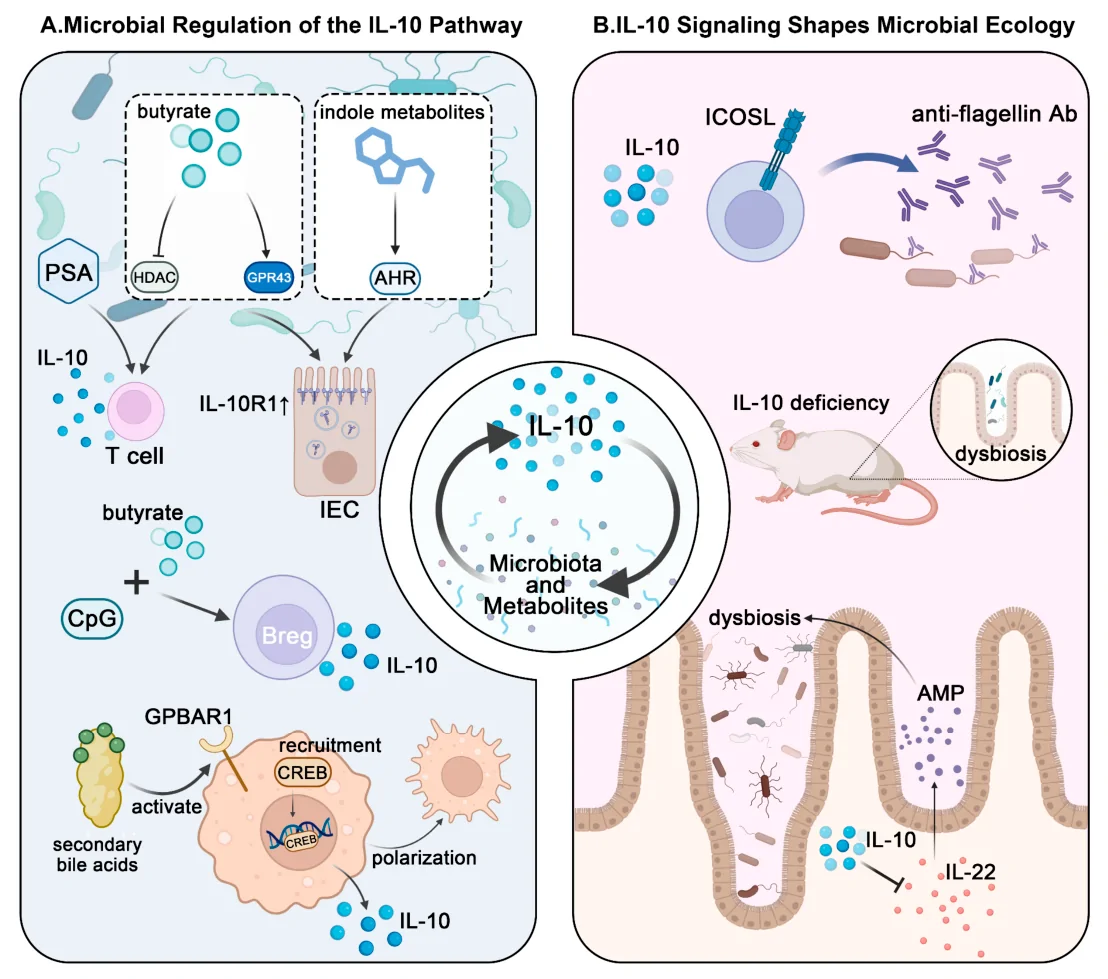
Reciprocal regulation between IL-10 signaling and the gut microbiota. (**A**) Microbial components boost IL-10 production via butyrate, indole, CpG, PSA, and GPBAR1/TGR5-CREB pathways. (**B**) IL-10 signaling preserves microbial diversity by limiting antimicrobial pressure. IL-10, interleukin-10; IL-10R, interleukin-10 receptor; HDAC, histone deacetylase; GPR43, G protein-coupled receptor 43; AHR, aryl hydrocarbon receptor; PSA, polysaccharide A; IEC, intestinal epithelial cells; CpG, cytosine-phosphate-guanine; Breg, regulatory B cells; GPBAR1, G protein-coupled bile acid receptor 1; CREB, cAMP response element-binding protein; ICOSL, inducible T cell costimulator ligand; AMP, antimicrobial peptide.

Microbial regulation of the IL-10 pathway has two main inputs. Diffusible metabolites—including short-chain fatty acids (SCFAs), tryptophan derivatives and bile-acid metabolites—primarily affect epithelial and lymphoid cells, while structural components and pattern-recognition signals act on myeloid cells, dendritic cells, and regulatory B cells. Many commensals provide both cues (Figure 3A).

#### 4.1.1. Metabolite-Mediated Reinforcement of IL-10 Signaling

SCFAs, especially butyrate, reinforce the IL-10 pathway at ligand and receptor levels. Butyrate promotes IL-10-producing T cells via histone deacetylase (HDAC) inhibition and G protein-coupled receptor 43 (GPR43) signaling, boosting mucosal immune tolerance (mouse, in vivo and in vitro) [69], and increases IL-10 production in CD4^+^ T cells through PI3Kγ/mTOR signaling (mouse, in vivo and in vitro) [70]. It also upregulates IL-10R1 in intestinal epithelial cells via HDAC inhibition and STAT3 signaling, improving barrier function (human cell lines) [71]. Thus, butyrate amplifies the IL-10 pathway from both the ligand-producing and receptor-responsive sides.

Beyond these signaling actions, SCFAs can also influence host cell metabolism. In differentiated microbiota-specific Th1 cells, SCFAs act through GPR43 to activate STAT3/mTOR and induce Blimp-1, thereby promoting IL-10[72]. Related SCFAs such as pentanoate can reprogram T cell metabolism by enhancing glycolysis and pyruvate oxidation, increasing acetyl-CoA availability for histone acetylation at the *IL10* promoter [73]. Butyrate-conditioned intestinal epithelial cells (IECs) undergo sustained Sat1-dependent polyamine reprogramming and release N1-acetylspermidine, which at least partially contributes to sustained T cell IL-10 production [74]. This provides a direct link to Section 3, where IL-10 itself reprograms macrophage lipid metabolism, including fatty acid desaturation and sphingolipid homeostasis [5], and where an oxidative metabolic shift has been linked to increased T cell IL-10 production [6]. Conversely, IL-10-mediated reprogramming of host lipid metabolism may shape the intestinal niche available to commensals. For example, the mucosal lipid environment maintained by IL-10 could provide a colonization or nutrient-acquisition advantage for specific commensals, although direct evidence for this hypothesis is currently limited. Future studies integrating lipidomics, metagenomics, and spatial metabolomics are needed to test these connections.

Tryptophan derivatives provide a second, distinct metabolite-based route. Indole and related derivatives activate AHR to increase epithelial IL-10 expression, promote goblet cell differentiation, and maintain the mucus barrier, including during aging-related barrier decline (mouse, in vivo) [75]. Indole-3-propionic acid (IPA) similarly upregulates epithelial IL-10R1 expression through AHR activation, improving experimental colitis by increasing epithelial responsiveness to IL-10 (mouse, in vivo; human cell lines and enteroids) [76]. However, conflicting evidence exists: a study in pigs did not significantly increase ileal IL-10 mRNA under LPS challenge, although IPA still exerted protective effects [77], suggesting that it may act through alternative anti-inflammatory pathways. This highlights the need for caution in extrapolating findings across species and for further mechanistic studies.

Bile acids contribute to innate immune metabolic programming by activating G protein-coupled bile acid receptor 1 (GPBAR1), which promotes CREB recruitment to the IL-10 promoter, driving an anti-inflammatory macrophage phenotype (mouse, in vivo; macrophage cell line) [78]. In addition, bile acids can regulate IL-10 through the farnesoid X receptor (FXR) and other pathways, although the specific mechanisms and bile acid species involved require further investigation. Not all bile acids have the ability to regulate IL-10, and the net effect depends on the bile acid composition and the cellular context. This extends microbiota-IL-10 regulation to innate immune cells.

#### 4.1.2. Structural Signals: TLRs, STING, and Commensal Strains

Structural microbial components also directly engage host pattern-recognition pathways. Regulatory B cell IL-10 production can require both butyrate and bacterial cytosine-phosphate-guanine (CpG) motifs; *Clostridium sporogenes*, which provides both signals, efficiently promotes regulatory B cells (Breg) differentiation (mouse, in vivo and in vitro) [79]. *Clostridium butyricum* induces IL-10-producing intestinal macrophages via TLR2/MyD88, with anti-colitic effects depending on macrophage-derived IL-10 (mouse, in vivo and ex vivo) [80]. *Bifidobacterium breve* enhances IL-10 and IL-27 production by CD103^+^ dendritic cells, inducing colonic Tr1 cells (mouse, in vivo and in vitro) [81]. *Lactobacillus johnsonii* activates CD206^+^ IL-10-producing macrophages via TLR1/2—STAT3 pathway (mouse and human cells) [82]. *Faecalibacterium prausnitzii* conditions human dendritic cells to promote Tr1-like T cell differentiation through TLR2/6/c-Jun N-terminal kinase (JNK) signaling and induction of IL-10, IL-27, CD39, and indoleamine 2,3-dioxygenase 1 (IDO-1) (human cells) [83]. Beyond canonical TLR signaling *Lacticaseibacillus rhamnosus* GG induces IL-10 production in intestinal monocytes through the stimulator of interferon genes (STING)/TANK-binding kinase 1 (TBK1)/NF-κB pathway (mouse, in vivo and in vitro) [84]. Microbial signals that modulate the IL-10 axis are categorized in Table 2.

Commensals modulate IL-10 signaling through diverse microbial and metabolic pathways, engaging epithelial, T, and myeloid cells via distinct cellular routes.

### 4.2. IL-10 Feeds Back: Preserving Diversity, Restraining Pathobionts

Microbial signals boost IL-10, which in turn shapes gut microbes. IL-10 deficiency alters microbial diversity and promotes pathobionts before severe inflammation (Figure 3B).

#### 4.2.1. Diversity Maintenance and Pathobiont Restraint

Germ-free IL-10-deficient mouse studies show IL-10 affects microbial ecology. After colonization, IL-10-deficient mice develop reduced diversity and expansion of *Proteobacteria* and *E. coli* (mouse, in vivo) [87], with microbial and gene changes preceding colitis (mouse, in vivo) [88]. Thus, IL-10 aids microbial stability before dominant inflammatory damage.

#### 4.2.2. Control of Antimicrobial Selection Pressure

IL-10 preserves microbial ecology in part by limiting antimicrobial selection pressure. In IL-10-deficient mice, excessive IL-22 drives antimicrobial peptide (AMP) expression and reduces microbial diversity; deletion of IL-22 reverses this dysbiosis (mouse, in vivo) [89]. Thus, IL-10 maintains microbial diversity not only by suppressing inflammation but also by restraining epithelial antimicrobial pressure.

#### 4.2.3. Resilience Under Infectious and Environmental Perturbation

IL-10 signaling supports microbial resilience under stress. In a whipworm infection model, deficiency of IL-10, IL-10R1, or IL-10R2 causes cecal dysbiosis, with expansion of *Enterococcus* and *Enterobacteriaceae*, a phenotype dependent on hematopoietic rather than epithelial IL-10 signaling (mouse, in vivo) [90]. Environmental perturbations provide further examples. Antibiotic-perturbed maternal microbiota can be vertically transmitted and exacerbate colitis in IL-10-deficient offspring (mouse, in vivo) [91]. Chemotherapy with oxaliplatin reduces *Lactobacillus* and *Bifidobacterium*, and microbiota from chemotherapy-exposed mice suppress macrophage IL-10 production; probiotic supplementation restores IL-10 and mitigates chemotherapy-associated toxicity (mouse, in vivo; mouse and human cells) [92]. Collectively, these observations position IL-10 within a resilience system that governs whether the microbiota recovers or shifts toward a disease-promoting state after perturbation.

#### 4.2.4. Cooperation with Adaptive Mucosal Immunity

IL-10-dependent microbiota control also engages adaptive immune pathways. Inducible T cell costimulator ligand (ICOSL) signaling supports antibody responses against commensal flagellins derived from mucus-associated *Lachnospiraceae*; combined deficiency of ICOSL and IL-10 causes a profound loss of commensal control and early-onset colitis (mouse, in vivo) [7]. IL-10 thus cooperates with T cell help, B cell responses, and antibody-mediated containment to maintain host–microbiota mutualism.

Defined commensal molecules further illustrate this cooperative logic. Polysaccharide A (PSA) from *Bacteroides fragilis* protects against pathogenic commensal-driven colitis by suppressing intestinal Th17 responses via IL-10-producing CD4^+^ T cells (mouse, in vivo and in vitro) [85]. Similarly, lymphoid-tissue-resident commensal bacteria induce dendritic-cell-derived IL-10 and ILC3-derived IL-22. IL-10 limits pro-inflammatory Th17 responses and promotes immune tolerance, whereas IL-22 supports their colonization niche (mouse, in vivo and in vitro) [93]. These findings demonstrate that commensals can actively induce IL-10-dependent tolerance to stabilize their own ecological position within host tissues.

Collectively, the evidence above supports a bidirectional loop of IL-10–microbiota interaction that reflects a core evolutionary logic: the host uses IL-10 to limit excessive inflammation and maintain a colonization niche for commensal bacteria, while commensals reinforce the IL-10 pathway through metabolic and structural signals to maintain immune tolerance [94]. This positive feedback stabilizes host–microbiota mutualism. Specifically, host immunity limits excessive inflammation to protect commensal diversity, and commensal bacteria reinforce the IL-10 pathway via metabolism (e.g., SCFAs, tryptophan derivatives, bile acid metabolites) to maintain immune tolerance. Loss of IL-10 thus impairs immune restraint while destabilizing the microbial ecosystem, suggesting that microbiota-based interventions may need to restore both microbial composition and the host’s mucosal IL-10 responsiveness.

Despite the accumulating evidence for IL-10–microbiota crosstalk, several caveats warrant consideration. Much of the evidence for IL-10-dependent microbial diversity and stability derives from IL-10-deficient mice, in which the observed dysbiosis may be confounded by concurrent intestinal inflammation, altered antimicrobial peptide expression, genetic background, housing conditions, and maternal transmission. Thus, direct IL-10-dependent ecological effects should be distinguished from dysbiosis secondary to uncontrolled inflammation, and model-specific taxonomic changes should not be generalized as a universal IL-10 signature across all contexts.

## 5. Context-Dependent Roles Across Intestinal Disease

**Figure 4:**
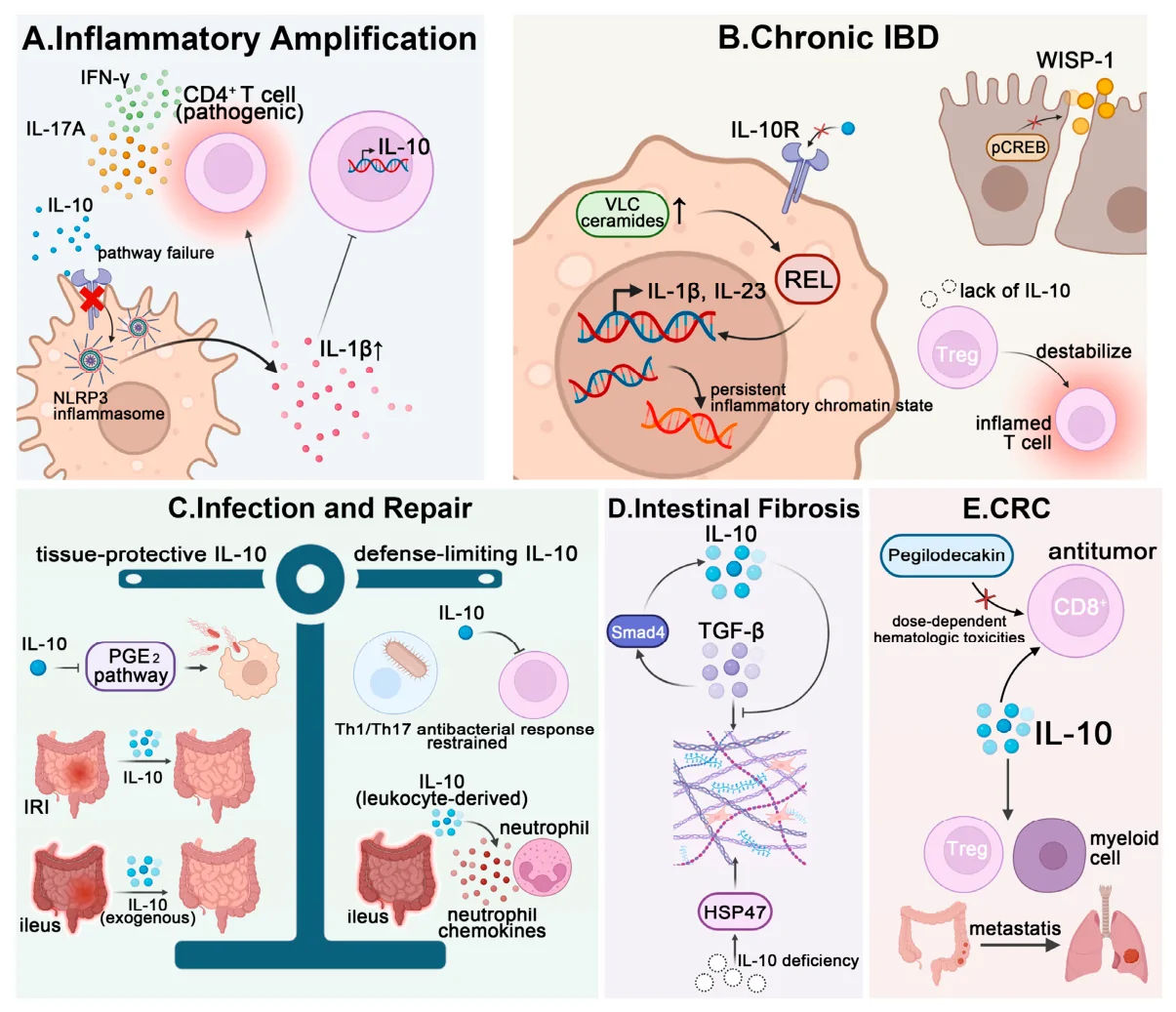
Context-dependent roles of IL-10 across intestinal diseases. (**A**) Inflammatory amplification: IL-10 pathway failure drives macrophage inflammasome activation, IL-1β production, and pathogenic CD4^+^ T cell responses. (**B**) Chronic IBD: impaired IL-10 signaling promotes VLC ceramide–REL inflammatory imprinting, stable chromatin alteration, defective phosphorylated CREB epithelial repair and Tregs destabilization. (**C**) Infection/injury: IL-10 protects by limiting barrier damage and PGE_2_ signaling, but may restrict Th1/Th17 antimicrobial responses or promote neutrophil-driven ileus depending on cellular source. (**D**) Fibrosis: IL-10 limits heat shock protein 47 (HSP47)-associated collagen deposition and counter-regulates TGF-β/Smad fibrotic remodeling. (**E**) CRC: IL-10 can enhance CD8^+^ T cell antitumor activity, while loss of CD8^+^ TIL-derived IL-10 (via complement) or Treg-derived IL-10 favors tumor progression and metastasis. IL-10, interleukin-10; IL-10R, interleukin-10 receptor; NLRP3, NOD-like receptor protein 3; IL-1β, interleukin-1 beta; IFN-γ, interferon gamma; IL-17A, interleukin-17A; VLC ceramides, very-long-chain ceramides; REL, Rel proto-oncogene, NF-κB subunit; WISP-1, WNT1-inducible signaling pathway protein 1; CREB, cAMP response element-binding protein; Treg, regulatory T cells; PGE_2_, prostaglandin E2; IRI, ischemia–reperfusion injury; Th1, T helper 1 cells; Th17, T helper 17 cells; TGF-β, transforming growth factor beta; HSP47, heat shock protein 47; CD8, cluster of differentiation 8; TIL, tumor-infiltrating lymphocyte; Treg, regulatory T cells.

IL-10 signaling is relevant in various intestinal disease states. Its effect varies by disease stage, cell type, inflammatory, microbial, and repair demand. This section covers IL-10 in inflammation onset, chronicity, infection, repair, and late complications (Figure 4).

### 5.1. Genetic and Acquired IL-10 Failure in VEO-IBD and Adult IBD

Loss-of-function in *IL10/IL10R* mutations causes severe infantile-onset IBD, and hematopoietic stem cell transplantation can induce remission, underscoring the central role of IL-10 signaling in disease pathogenesis (human, genetic analysis and clinical intervention) [3]. Common *IL10* variation is also associated with UC susceptibility (human, GWAS) [95], indicating that both rare high-penetrance defects and common risk alleles converge on this pathway. More recently, neutralizing anti-IL-10 autoantibodies have been identified in infantile IBD, revealing an additional mechanism of functional IL-10 deficiency (human, case report) [96].

These genetic and acquired defects converge on macrophage-centered inflammatory amplification. In the absence of intact IL-10 signaling, macrophages fail to restrain inflammasome activity and overproduce IL-1β, which then drives pathogenic CD4^+^ T cell production of IFN-γ and IL-17A (mouse, in vivo; mouse and human cells) [30] (Figure 4A).

Macrophages thus represent a critical IL-10-responsive population: IL-10R signaling conditions intestinal macrophages toward an anti-inflammatory state required for mucosal homeostasis (mouse, in vivo; mouse and human cells) [97], and macrophage-restricted deletion of IL-10R is sufficient to cause severe spontaneous colitis, even when macrophage-derived IL-10 itself is preserved (mouse, in vivo and ex vivo) [11].

This principle extends to neonatal intestinal disease. In necrotizing enterocolitis (NEC), IL-10 deficiency aggravates epithelial injury and inflammation, whereas maternal milk increases intestinal IL-10 and reduces disease severity (mouse and rat, in vivo) [98,99]. Although NEC differs from IBD in developmental timing and pathophysiology, these observations support a shared concept: early-life intestinal homeostasis critically depends on IL-10-mediated restraint of inflammatory and barrier-damaging responses.

Consistently, anti-TNF therapy, a mainstay of IBD treatment, induces macrophage IL-10 production (mouse and human cells) [55], indicating IL-10 restoration as an integral component of its efficacy.

### 5.2. Chronic IBD: Metabolic Scars and Barrier Breakdown

Once intestinal inflammation becomes chronic, IL-10 pathway dysfunction contributes more as a failure to restore tissue homeostasis. Two processes are especially relevant: persistent inflammatory metabolic programming in immune cells and defective barrier repair (Figure 4B).

At the metabolic level, IL-10 deficiency permits accumulation of saturated very-long-chain ceramides and sustained REL-dependent inflammatory gene expression in macrophages (mouse, in vivo and ex vivo) [5]. In parallel, it causes stable chromatin alterations in intestinal macrophages that are not fully reversed by later IL-10 exposure (mouse, in vivo and ex vivo) [52]. Prolonged IL-10 insufficiency may therefore imprint macrophages with a persistent inflammatory state, helping explain why chronic intestinal inflammation can resist reversal even after partial cytokine blockade.

At the tissue level, loss of IL-10 impairs epithelial repair and simultaneously destabilizes Tregs within inflamed sites (mouse, in vivo and ex vivo) [8,44], thereby weakening both barrier restoration and immune regulation.

Human IBD further illustrates that chronic disease can involve both impaired IL-10 production and impaired IL-10 responsiveness. In Crohn’s disease (CD), a defect in a Notch/STAT3-driven Blimp-1/c-MAF molecular switch limits IL-10 induction in effector CD4^+^ T cells, favoring a Th1/Th17 inflammatory phenotype (human cells) [100]. Antigen-specific defects have also been reported, with *Escherichia coli OmpC*-specific CD4^+^ T cells from CD patients showing reduced IL-10 production (human cells) [101]. Moreover, IL-10-producing CD4^+^ T cells are functionally heterogeneous, and only Foxp3^−^ subsets co-expressing specific inhibitory receptor combinations exhibit strong regulatory activity, and loss or dysfunction of these subsets may further erode effective immune regulation in IBD (mouse and human cells) [102].

Collectively, these findings suggest that chronic intestinal inflammation is sustained by combined defects in metabolic resolution, epithelial repair, and regulatory-cell stability. These layered failures may limit the benefit of simply increasing IL-10 levels once inflammatory programs are established.

### 5.3. Infection and Injury: The Protective-Brake Paradox

During intestinal infection or acute tissue injury, IL-10 acts as a protective brake limiting immunopathology, but this restraint can also reduce pathogen clearance. Its role depends on the balance between tissue protection and antimicrobial immunity (Figure 4C).

In sterile or noninfectious tissue injury, the protective role of IL-10 is clear. As noted above, macrophage-derived IL-10 promotes epithelial WISP-1 signaling and wound closure after mucosal injury (mouse, in vivo; human enteroids) [8]. IL-10-deficient mice show higher mortality, intestinal damage, and inflammation after ischemia–reperfusion injury (IRI) (mouse, in vivo) [103]. These findings highlight IL-10’s role in limiting secondary inflammatory injury during epithelial damage.

Postoperative ileus shows IL-10’s context-dependent role in tissue injury. Early studies found IL-10-deficient mice had poor recovery and high mortality after intestinal manipulation, while exogenous IL-10 reduced inflammation and motility issues (mouse, in vivo and ex vivo) [104]. Later research indicated leukocyte-derived IL-10 worsened ileus by promoting neutrophil activity, and its deletion reduced severity (mouse, in vivo; human, clinical) [105]. These conflicting results highlight differences in injury models, IL-10 sources, timing, and pathways, cautioning against assuming IL-10 is uniformly protective in intestinal injury.

In helminth infection, IL-10 is needed to balance immune responses, and its deficiency leads to severe intestinal damage and increased mortality (mouse, in vivo) [106]. Mechanistically, IL-10 signaling limits IFN-γ and supports Th2 responses, reducing immunopathology (mouse, in vivo and in vitro) [107]. During *Citrobacter rodentium* infection, IL-10 deficiency attenuates Th1/Th17 responses, speeds pathogen clearance, and reduces colitis; transient immune activation can protect IL-10-deficient mice from later spontaneous colitis (mouse, in vivo and ex vivo) [108]. However, IL-10 signaling is not purely anti-antimicrobial. In macrophages, loss of IL-10R impairs intracellular killing of *Salmonella* by overactivating the prostaglandin E_2_ (PGE_2_) pathway, indicating that IL-10 can preserve innate effector quality under specific infectious conditions (human, ex vivo) [109].

Thus, IL-10 is a context-dependent regulator of host defense, not just a generic suppressor of antimicrobial immunity. It protects tissues from damage, but its effect on pathogen clearance varies by pathogen class, infected cell type, inflammatory timing, and dominant effector pathway.

### 5.4. Fibrosis and Cancer: Divergent Outcomes of Tissue Remodeling

In late-stage tissue remodeling, the consequences of IL-10 signaling diverge markedly between fibrosis and cancer, dictated largely by cellular source and target-cell identity.

In intestinal fibrosis, IL-10 primarily acts as an endogenous brake on excessive scarring (Figure 4D). IL-10-deficient mice show increased collagen deposition and fibrosis during experimental colitis and after ileocolonic anastomosis, accompanied by stronger Th1 responses to bacterial antigens (mouse, in vivo) [110,111]. IL-10 also counter-regulates transforming growth factor beta 1 (TGF-β1)-driven fibrosis: TGF-β1 induces IL-10 via Smad-dependent mechanisms, and IL-10 in turn attenuates TGF-β1-mediated fibrotic activity (mouse, ex vivo) [112], establishing a negative-feedback loop that limits pathological repair.

In CRC, IL-10 biology is context-dependent and not straightforwardly translatable. On one hand, IL-10 can enhance antitumor immunity by promoting CD8^+^ T cell expansion and effector function; pegylated IL-10 (pegilodecakin) showed promising single-arm activity in combination with immune-checkpoint blockade in early clinical exploration, but this approach has been limited by dose-dependent hematologic toxicities (thrombocytopenia, anemia) and a negative randomized Phase 3 SEQUOIA trial in pancreatic cancer (OS 5.8 vs. 6.3 months; no improvement) [10,113]; oral sustained-release IL-10 suppressed colon carcinogenesis in preclinical models (mouse, in vivo) [114]. On the other hand, Foxp3^+^ Treg-derived IL-10 can promote metastasis by acting on myeloid cells and Tregs (mouse, in vivo) [115]. Whether IL-10 agonism helps or harms a given tumor is therefore governed by cellular source, target compartment, and stage, and no IL-10-directed strategy has been established as a CRC therapy in humans. This duality explains why systemic IL-10 agonism has faced translational limitations: it cannot reliably restrict signaling to antitumor effector cells while avoiding regulatory compartments.

Newer engineering strategies address this limitation by spatially biasing IL-10 delivery. An anti-programmed cell death protein 1 (PD-1)–IL-10 fusion protein delivers attenuated IL-10 activity preferentially to PD-1^+^ T cells, strengthening antitumor responses in PD-1-resistant models (mouse and non-human primate, in vivo) [116], while IL-10/Fc enhances terminally exhausted CD8^+^ T cell function via oxidative phosphorylation (mouse, in vivo; mouse and human cells) [117]. Functional heterogeneity among IL-10-producing CD4^+^ T cell subsets and genetic variation in IL-10R1 further shape these outcomes (mouse and human cells) [102,118].

Thus, in fibrosis IL-10 primarily restrains excessive matrix deposition, whereas in cancer it can either enhance CD8^+^ T cell immunity or support immunosuppressive networks. Therapeutic targeting therefore requires directing IL-10 signaling to the appropriate cell type and disease stage.

## 6. Therapeutic Strategies: From Replacement to Precision Tuning

**Figure 5:**
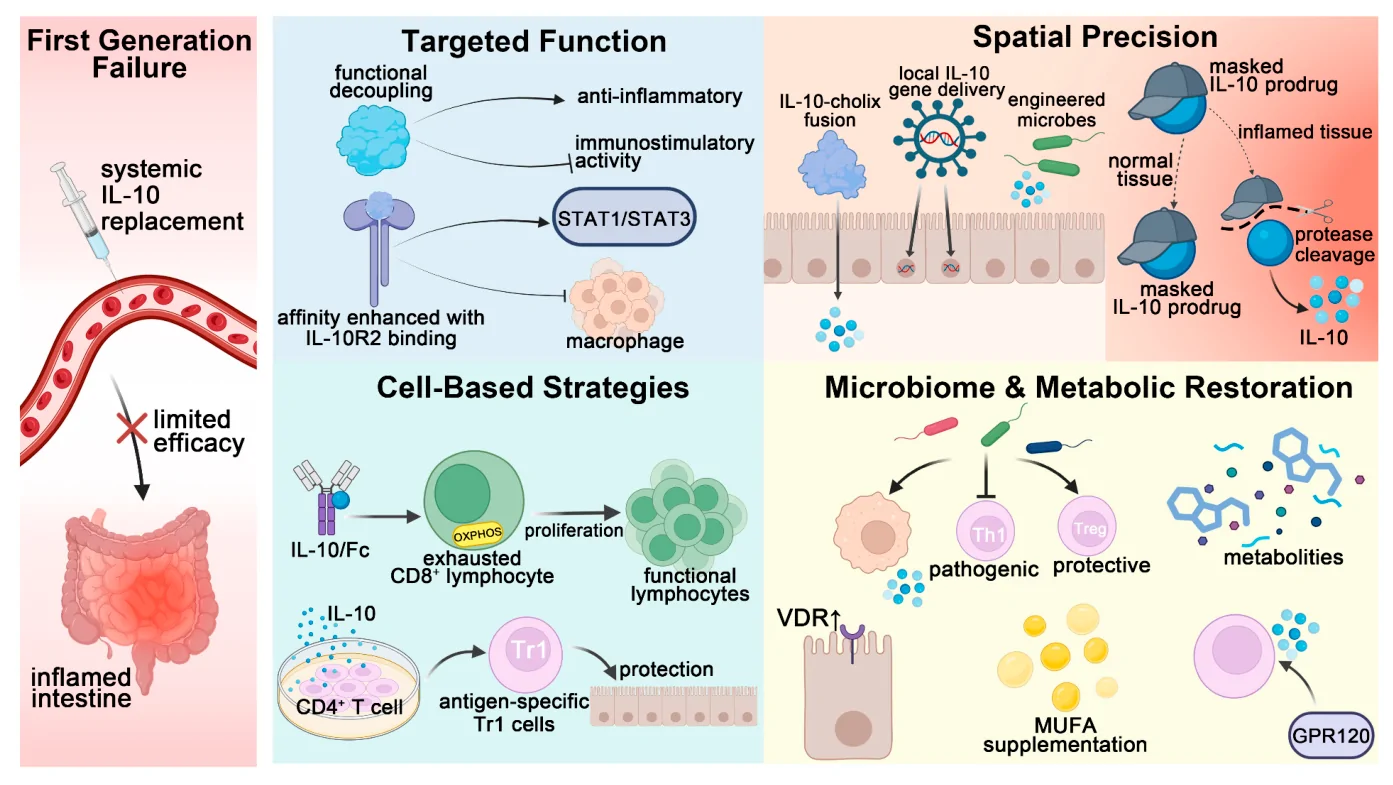
Precision strategies to restore IL-10-mediated intestinal protection. Systemic replacement failed due to poor localization and cell specificity. Current precision approaches—gut-targeted delivery, molecular engineering, cell-based regulation, and microbial or metabolic support—aim to restore localized, cell-selective IL-10 activity and endogenous responsiveness. IL-10, interleukin-10; IL-10R, interleukin-10 receptor; OXPHOS, oxidative phosphorylation; Tr1, type 1 regulatory T cells; Treg, regulatory T cells; VDR, vitamin D receptor; MUFA, monounsaturated fatty acid; GPR120, G protein-coupled receptor 120.

First-generation recombinant human IL-10 failed to induce clinical remission superior to placebo in CD [119]. This failure is best understood as a mismatch between systemic ligand replacement and the spatially restricted, cell-specific, and metabolically conditioned biology of endogenous IL-10, rather than as evidence against the pathway itself [120]. Additional factors may include differences in the biophysical properties of recombinant versus locally produced endogenous IL-10 (e.g., glycosylation, aggregation, receptor binding kinetics, and tissue penetration), as well as unknown variables. Thus, the failure of systemic IL-10 should not be attributed to a single mechanism. The trial program also revealed a narrow therapeutic window and other limitations examined in Section 6.1[119,121]. This realization has shifted therapeutic development from simple replacement toward selective restoration of IL-10 signaling in defined tissue and cellular compartments (Figure 5). Nevertheless, the possibility that biophysical differences between recombinant and endogenous IL-10 contributed to the failure should be considered in future trial design.

### 6.1. Why Systemic IL-10 Failed: A Mismatch of Scale and Space

The failure of systemic IL-10 in CD reflects a fundamental mismatch between exogenous delivery and endogenous biology. Systemic administration has a narrow therapeutic window: doses produced only modest, dose-dependent, and reversible decreases in hemoglobin and platelet counts (human, clinical trial) [121], and cannot replicate the local, cell-specific actions of endogenous IL-10 [44]. As discussed in Section 2.4, inflamed tissues can become hyporesponsive to IL-10. In addition, prolonged IL-10 deficiency imprints macrophages with stable inflammatory chromatin states [52], and inflamed tissues can become functionally resistant via Shp2 upregulation or microbial stimulation [37,38]. Moreover, systemic delivery cannot selectively engage protective compartments, because IL-10 exerts divergent effects depending on the target cell: it suppresses pathogenic Th17 responses via macrophages (mouse, in vivo and ex vivo) [122], while enhancing CD8^+^ T cell antitumor function (mouse, in vivo and ex vivo) [117]. Systemic delivery thus simultaneously risks activating beneficial and undesirable circuits. Genetic *IL10/IL10R* defects, reviewed in Section 5.1, confirm that the pathway itself is non-redundant for mucosal homeostasis [3,4]. Thus, the failure was not a failure of the pathway but a failure of pharmacological control over where, in which cell, and under what metabolic state IL-10 signals. Spatial mismatch and acquired cellular hyporesponsiveness are therefore plausible contributors to be tested.

### 6.2. Gut-Restricted Delivery: Enforcing Spatial Precision

Gut-restricted strategies aim to concentrate IL-10 activity in the diseased intestine. AMT-101, an IL-10-cholix fusion protein, exploits epithelial transcytosis to deliver IL-10 into the lamina propria with low systemic exposure and showed acceptable tolerability in early ulcerative colitis studies [123,124]. However, the Phase 2 LOMBARD trial (NCT04583358) in UC [125] did not meet its primary endpoint: clinical remission was 17.1% (12/70) with AMT-101 versus 20.0% (7/35) with placebo [126]. The Phase 2 MARKET trial (AMT-101 plus adalimumab) also missed its primary endpoint (remission 31.8% vs. 33.3% with adalimumab alone), although a post hoc subgroup of patients with disease duration < 5 years showed a numerical benefit (43.8% vs. 15.4%) [127]. These negative results indicate that gut-restricted delivery alone does not guarantee efficacy and that patient stratification is likely required. Intrarectal lentiviral IL-10 delivery achieves sustained local expression and suppresses colitis, though integrating vectors raise safety concerns [128]. Engineered probiotics, such as stress-inducible *Lactococcus lactis* or thymidylate synthase-deleted strains, provide a colonizing delivery platform [129,130,131,132]. WTX-921, a protease-activated IL-10 prodrug, releases active IL-10 in inflamed colonic tissue, coupling activation to pathology [133].

### 6.3. Engineered Variants: Biasing the Signal to Desired Responders

Given the pleiotropy of IL-10, therapeutic variants should engage protective responder populations while avoiding undesirable immunoregulation. An early Ile87Ala substitution reduced immunostimulatory activity while preserving anti-inflammatory properties [134]. Structure-guided engineering variants with altered IL-10R2 binding preferentially suppress macrophages over CD8^+^ T cells [24], while affinity-enhanced ones stabilize signaling for stronger STAT1/STAT3 activation at low concentrations [135]. Fusion proteins further combine signaling bias with targeting advantages: GFT-IL10M, an IgG-scaffolded monomer, biases activity toward monocytes [136], and an attenuated IL-10 monomer fused to anti-PD-1 delivers IL-10 in cis to PD-1^+^ T cells [116]. This construct, designated FP008, is currently in a Phase 1 trial (NCT06990698) that is recruiting participants; no clinical efficacy data have been reported to date [137]. These approaches shift therapy from cytokine supplementation to signal design.

### 6.4. Cell-Based Approaches: Rebuilding Endogenous Regulatory Circuits

Restoring endogenous regulatory circuits may approximate physiological IL-10 delivery. Prolonged activation of CD4^+^ T cells with IL-10 generates Tr1 cells with antigen-specific suppressive activity [138], and microbiota-specific Th17 cells can acquire a c-MAF-dependent IL-10^+^ regulatory phenotype in the lamina propria [20], the key challenge is functional stability in inflamed tissue. Early phase exploratory studies of Tr1 cell therapy have been reported [139], but the later Ovasave Phase IIb program (NCT02327221) was terminated [140], underscoring the challenges of manufacturing, functional stability, and persistence in inflamed tissues. Metabolic preconditioning provides one potential solution: in macrophages, mitochondrial superoxide and PKM2 enhance IL-10 production [53,54]; in T cells, DCA boosts oxidative metabolism, upregulates IL-10 via AHR/Bhlhe40, and enables Th1 cell-mediated suppression in colitis [6]. IL-10/Fc also enhances exhausted CD8^+^ T cell function through OXPHOS [117]. Effective cell therapy thus requires metabolic fitness, antigen specificity, and lineage stability.

### 6.5. Microbiome and Metabolic Interventions: Restoring Responsiveness from Outside

Microbial and metabolic interventions engage IL-10 biology through distinct routes that must not be conflated: (i) IL-10-dependent enhancers that boost endogenous IL-10 production or IL-10R responsiveness (e.g., butyrate, indole metabolites); (ii) interventions that may restore target-cell competence without requiring IL-10 ligand (e.g., metabolic preconditioning of macrophages); and (iii) IL-10-independent compensatory pathways that ameliorate colitis in IL-10-deficient mice but do not represent IL-10 signal restoration (e.g., *Alistipes timonensis* outer-membrane vesicles remodeling lipid profiles [141]; epithelial vitamin D receptor (VDR) expression [142]).

Select adherent *E. coli* strains increase IL-10-producing macrophages and protective Tregs, and suppress colitis through macrophage and epithelial IL-10R signaling [143,144] (class i), though strain-level precision is critical. Defined microbial products offer greater control: peptidoglycan from *Bifidobacterium adolescentis* induces regulatory B cells via TLR2 [86] (class i). Outer membrane vesicles from *Alistipes timonensis* remodel lipid profiles and delay colitis in IL-10-deficient mice [141] (class iii); and the tryptophan metabolite indole-3-carbaldehyde promotes barrier repair via AHR/IL-10/Wnt signaling [145] (class i). Host metabolic pathways are also targetable: MUFA supplementation may compensate for IL-10-dependent sphingolipid dysregulation [5] (class ii); epithelial VDR expression ameliorates colitis in IL-10-deficient mice [142] (class iii); and GPR120 agonism drives CD4^+^ T cell IL-10 production through Blimp-1/mTOR-dependent glycolysis, protecting against colitis [146] (class i). These interventions engage endogenous networks but lack specificity; future development requires biomarkers to distinguish protective IL-10 restoration from nonspecific immunosuppression, and a comparison of emerging therapeutic strategies is provided in Table 3.

## 7. Remaining Challenges and Path Forward

The role of IL-10 in intestinal immune homeostasis depends on factors like distribution, target cells, receptors, signaling, metabolism, and hyporesponsiveness, not just abundance, complicating interpretation and therapy.

### 7.1. Translational Gap Between Animal Models and Human Disease

**Figure 6:**
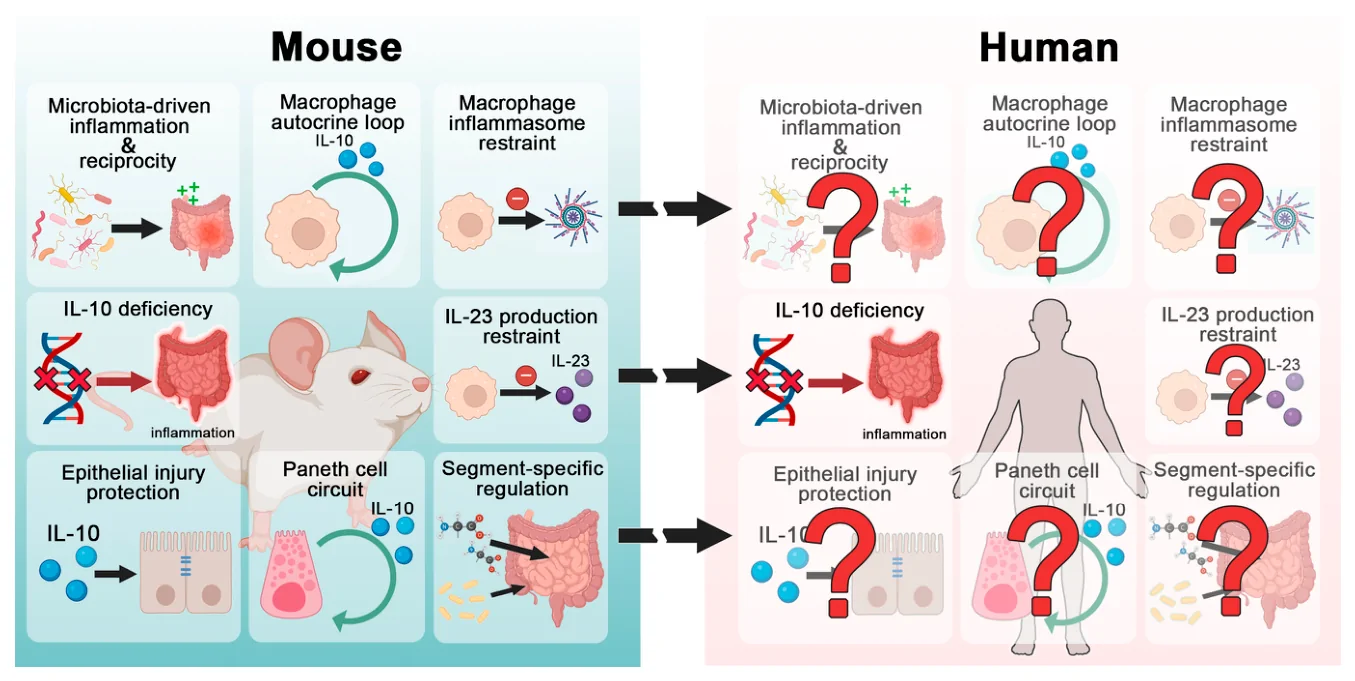
The translational gap between animal models and human disease. (**Left panel**) established IL-10-dependent protective mechanisms in mice, including microbiota–host reciprocity, macrophage autocrine IL-10 signaling (restraining IL-23 and inflammasome activation), genetic IL-10-deficiency-driven colitis, epithelial protection, Paneth cell autocrine circuit, and segment-specific macrophage regulation. (**Right panel**) corresponding evidence in humans. Red question marks denote mechanisms unconfirmed or unresolved in human systems; only inactivating *IL10/IL10R* mutations causing severe early-onset IBD are genetically validated. IL-10, interleukin-10; IL-23, interleukin-23.

Much of the current framework for intestinal IL-10 biology has been established in mouse models, especially *IL10^−/−^* and *IL10R*-deficient systems, which models have been indispensable for defining the requirement for IL-10 in restraining microbiota-driven inflammation [149], macrophage inflammasome activation [42], IL-23 production [29], and epithelial injury [59]. These models, however, do not fully recapitulate human intestinal disease, and several conclusions drawn from them require explicit validation in human systems before they can anchor clinical translation. The comparison below contrasts the principal IL-10 mechanisms by the strength of mouse versus human evidence, Table 4 provides the summary of the translational gap between animal models and human disease. Figure 6 provides a visual synthesis of this translational gap, contrasting the robust IL-10 mechanisms established in murine systems with the narrower human evidence base and the persistent therapeutic shortfall.

Human genetics provides the strongest validation of IL-10 necessity: inactivating *IL10/IL10R* mutations cause severe, early-onset, refractory colitis, establishing IL-10 signaling as a non-redundant requirement for human mucosal immune regulation [3,4]; this aligns with and extends the mouse loss-of-function phenotype [149]. By contrast, cellular and spatial mechanisms are largely mouse-supported. The closest human parallel to the murine macrophage autocrine loop is the demonstration that Shp2 up-regulation disrupts IL-10—STAT3 signaling in macrophages from IBD patients [37], yet a direct human autocrine IL-10—IL-23 circuit has not been shown. Segment-specific regulation [18,19] and the proposed Paneth-cell circuit [15] remain unconfirmed in human intestine, and microbiota reciprocity is confounded in mice by inflammation, antimicrobial peptides, genotype, housing, and maternal transmission [91], so human associations should be read as correlative rather than causal.

The translational gap is most sharply exposed by therapeutics. Despite consistent preclinical efficacy, first-generation recombinant human IL-10 failed to induce remission superior to placebo in CD, with a narrow therapeutic window and dose-dependent, reversible anemia and thrombocytopenia [121]. More recently, gut-restricted AMT-101 showed acceptable tolerability in early UC studies [124] but missed its primary endpoints in the Phase 2 LOMBARD (clinical remission 17.1% vs. 20.0% with placebo [126]) and MARKET trials [127]. Preclinical promise has thus repeatedly failed to predict human efficacy, underscoring that mouse-derived mechanisms are necessary but insufficient guides to therapy.

Bridging this gap will require human organoids, organ-on-chip platforms, and immune-epithelial-microbial co-cultures, together with spatial and single-cell mapping of human mucosal biopsies (Section 7.2) and biomarker-guided trial designs (Section 8.3). The translational gap outlined above is summarized schematically in Figure 6, which contrasts the murine-established IL-10 mechanisms (left) with the human evidence base (right) and highlights where preclinical promise has not predicted therapeutic success.

### 7.2. Spatial and Cellular Heterogeneity: Unresolved Architecture

IL-10 functions as a compartmentalized paracrine regulator, yet the spatial architecture of its sources and targets in human intestine remains poorly resolved. In mice, separate studies of small-intestinal and colonic macrophages suggest a segment-specific ‘anatomical logic’ for IL-10 sources (diet-dependent in the small intestine [18]; commensal-dependent in the colon [19]); however, this organization is inferred from non-spatial, largely mouse, studies and has not been confirmed by direct spatial mapping in human intestine, where such evidence is currently limited.

A practical consequence is the abundance-efficacy paradox in IBD: elevated mucosal IL-10 coexists with persistent inflammation [41,43], which may reflect spatial segregation of IL-10 sources from IL-10R-expressing responders, insufficient receptor signaling, or acquired hyporesponsiveness [39,40] rather than a simple ligand deficit. Bulk transcriptomics and whole-biopsy cytokine measurements cannot resolve these couplings, and most human data to date are limited to bulk biopsy cytokine quantification or single-cell RNA sequencing without spatial coordinates; even where p-STAT3^+^ cells have been identified in human IBD mucosa, they have not been co-localized with IL-10-producing sources.

Resolving the architecture will require spatially resolved approaches: spatial transcriptomics, multiplexed imaging mass cytometry or CODEX, and in situ co-detection of IL-10^+^ source cells, IL-10R-expressing targets, and p-STAT3-positive responders within intact human mucosal architecture across disease states. Until such human spatial data are available, claims about human IL-10 spatial organization should be framed as model-derived hypotheses rather than established anatomy.

### 7.3. Reversibility and Biomarker Gaps

IL-10 dysfunction can leave durable metabolic and epigenetic scars. In macrophages, IL-10 deficiency drives persistent inflammatory chromatin states [52] and pathological sphingolipid accumulation [5,150,151] that are not fully reversed by later IL-10 exposure [52]; MUFA supplementation may partially compensate [5]. It remains unclear when impaired signaling is reversible and when the tissue has entered a fixed inflammatory state. This uncertainty is compounded by the absence of validated biomarkers to define IL-10 pathway competence beyond cytokine abundance. Elevated mucosal IL-10 despite persistent inflammation [39,41], high Shp2 expression [37], persistent IL-23 production [38], or macrophage inflammatory imprinting [52] may indicate a need to restore responsiveness rather than increase ligand. Among these, impaired STAT3 phosphorylation—directly demonstrated in IBD macrophages with Shp2 upregulation [37]—serves as a more definitive indicator of IL-10 hyporesponsiveness. Candidate biomarkers (IL-10R [152], STAT3 phosphorylation [37], Shp2 [37,153], sphingolipids [154,155], microbial metabolites [156]) have been identified in exploratory studies but lack prospective clinical validation. Without companion diagnostics, IL-10-based interventions risk repeating the failure of systemic recombinant IL-10 in unstratified populations [121].

### 7.4. Therapeutic Precision Does Not Eliminate Safety Concerns

Precision strategies that prolong signaling, bias receptor engagement, or restrict activation to inflamed tissue [24,133,136,157] may improve efficacy but introduce new safety concerns. In cancer, IL-10 can either enhance CD8^+^ T cell antitumor function or promote Treg-mediated metastasis [10,115,116,117]; in infection, IL-10 limits tissue damage but may restrain protective antimicrobial responses in a pathogen- and timing-dependent manner [106,107,108,109]. Long-term considerations include the theoretical risk of sustained STAT3 activation with chronic IL-10 exposure [63], altered epithelial proliferation [75], context-dependent effects on tumor immunity [158], and the fact that loss of IL-10 signaling can itself impair bacterial killing [109]—arguing for tightly localized, biomarker-guided delivery rather than systemic agonism. These limitations indicate that IL-10-directed therapy should be matched to the dominant form of pathway dysfunction in a given tissue—insufficient production, impaired delivery, acquired hyporesponsiveness, or fixed inflammatory imprinting.

## 8. Outlook and Future Directions

To harness IL-10 therapeutically, it must be understood as a spatially organized, cell-specific, and metabolically conditioned homeostatic pathway. The following priorities are critical.

### 8.1. Human Tissue Map of IL-10 Action: Where, Who, and Why It Fails

A major goal is to map IL-10 production, sensing, and signaling failure across human intestinal compartments (epithelial, myeloid, stromal, and lymphoid) by integrating source cells, IL-10R-expressing targets, STAT3 activation with anatomical localization across disease states [159]. Such a map would resolve whether elevated mucosal IL-10 in IBD represents effective counter-regulation or a failed compensatory response in IL-10-refractory tissue [39,40], distinguishing poor ligand access, insufficient receptor availability, defective downstream signaling, and spatial uncoupling between source and responder cells.

### 8.2. Deficiency vs. Hyporesponsiveness: Two Faces of IL-10 Dysfunction

IL-10 deficiency (inadequate local production or delivery) and acquired hyporesponsiveness (poor target-cell responsiveness despite abundant IL-10) require distinct interventions. Strategies to restore local IL-10 production or delivery are reviewed in Section 6; hyporesponsiveness is more likely to require combination strategies that restore target-cell competence—correcting lipid imbalance, reducing mitochondrial stress, or reversing chromatin imprinting [5,42]. Patient-derived macrophages, organoids, and ex vivo mucosal cultures should be used to test whether a given approach restores IL-10 responsiveness, not merely suppresses inflammation.

### 8.3. Biomarker-Guided Trials: Moving Beyond IL-10 Abundance

Building on Section 7.3, candidate biomarkers of IL-10 pathway competence have been identified but not prospectively validated. Future trials should therefore stratify patients using a composite functional index rather than IL-10 abundance alone, and pair clinical endpoints with pharmacodynamic readouts from paired mucosal sampling and ex vivo pathway testing. Adaptive designs should test whether such stratification improves responses to compartment-directed IL-10 therapies (Section 6).

### 8.4. Four Testable Hypotheses for Next-Generation IL-10 Therapy

To convert the above framework into testable, disease-anchored predictions for IBD, we propose four hypotheses, each linked to a defined methodological approach.

**Hypothesis** **1.** *Spatial mismatch. IL-10-producing cells and p-STAT3^+^ responder cells are spatially uncoupled in active IBD, and the severity of this uncoupling correlates with inflammation and poor therapy response. Test: spatial transcriptomics plus multiplex immunohistochemistry/CODEX on paired IBD biopsies; quantify the distance between IL-10^+^ sources and p-STAT3^+^ targets and correlate with endoscopic/clinical scores and anti-TNF response. Prediction: patients with tight source-responder coupling will respond better to IL-10-restorative strategies.*

**Hypothesis** **2.** *Metabolic-epigenetic reversibility. Restoring macrophage lipid homeostasis (e.g., correcting MUFA synthesis or limiting pathological sphingolipid accumulation [5]) can reverse inflammatory chromatin states and recover IL-10 responsiveness. Test: ex vivo IBD macrophages treated with lipid interventions, assessed by ATAC-seq/chromatin accessibility and p-STAT3 readout. Prediction: reversible (not fixed) imprinting predicts response to metabolic-restorative rather than ligand-addition therapy.*

**Hypothesis** **3.** *Biomarker stratification. A composite mucosal index combining IL-10R expression, STAT3 phosphorylation, Shp2 [37], sphingolipids [5], and microbial metabolites can prospectively predict response to IL-10 supplementation versus metabolic-restorative therapy. Test: prospective cohort with paired baseline/treatment biopsies; correlate the composite index with clinical and pharmacodynamic response. Prediction: biomarker-stratified enrollment improves response versus unstratified populations and would have avoided repeating the first-generation rhIL-10 failure in CD (see Section 6 and Section 7.3 for rationale).*

**Hypothesis** **4.** *Compartment-selective delivery. Therapeutic benefit depends on signaling restoration in the relevant compartments (macrophages, epithelial cells, Tregs) rather than indiscriminate exposure. Test: compare gut-restricted delivery (AMT-101 [124]), engineered variants [24,135], and microbial/metabolic interventions [75,147,149] in human tissue platforms and stratified trials. Prediction: compartment-matched delivery outperforms systemic exposure and explains why anatomically precise strategies may succeed where systemic supplementation failed (see Section 6 for limitations of systemic delivery).*

These hypotheses are deliberately tied to therapeutic outcome: each specifies not only a mechanism but also a measurable biomarker or spatial readout that would distinguish a responsive from a refractory tissue, thereby converting the review’s framework into an actionable roadmap for next-generation IL-10 therapy.

### 8.5. The Realistic Goal: Restoring Compartmentalized Competence

The objective is not to globally increase IL-10 levels, but to restore the capacity of selected mucosal compartments to use IL-10 appropriately. As discussed in Section 6 and Section 7, the therapeutic value of IL-10 depends on spatial delivery, target-cell competence, metabolic context, and disease stage. Future therapies should therefore be judged by whether they restore localized, cell-appropriate signaling rather than by systemic ligand exposure alone. Failure of one IL-10-directed strategy does not imply failure of the pathway: local deficiency and acquired hyporesponsiveness are distinct forms of dysfunction and will likely require distinct interventions (Section 8.2), as reflected in the hypotheses in Section 8.4.

## 9. Conclusions

IL-10 is essential for intestinal homeostasis, but its therapeutic value cannot be reduced to immunosuppression or ligand abundance. Rather than acting as a simple anti-inflammatory cytokine, IL-10 functions as a spatially organized, cell-type-specific, and metabolically conditioned pathway that integrates immune regulation, epithelial protection, and host–microbiota mutualism. The failure of systemic IL-10 supplementation in CD therefore reflects the limits of ligand replacement, not of the IL-10 pathway itself. Future strategies should restore compartment-appropriate IL-10 signaling and target-cell responsiveness through spatially and functionally controlled delivery, engineered variants, cell-based approaches, and microbiome- or metabolism-directed interventions. Achieving this will require human spatial mapping and composite biomarkers that distinguish insufficient local delivery, acquired IL-10 hyporesponsiveness, and fixed inflammatory imprinting, so that next-generation IL-10 therapies can be tested in appropriately stratified patients.

## Figures and Tables

**Table 1:** Cell-type-specific responses to IL-10 in the intestine.

Cell Population	IL-10 Role	Main Mechanism/Outcome	Evidence Level	Refs
Macrophage	Responder (also a source)	Suppresses NF-κB inflammatory genes; restricts NLRP3/IL-1β; autocrine control of IL-23 in colon	Mouse; human explants	[5,29,30,42]
Dendritic cell	Responder	Interferes with IRF transcriptional programs; downregulates CD86 (reduced antigen presentation)	Mouse; human in vitro	[28,43]
Treg	Responder (source)	STAT3-dependent Foxp3 stability; suppresses Th17	Mouse; human Treg in vitro	[32,33,44]
Th17	Responder	Attenuates Th17 differentiation; limits pathogenic responses (direct RORγt inhibition not formally shown)	Mouse; in vitro	[31,33]
Intestinal epithelial cell	Responder	Limits ER stress, apoptosis; upregulates ZO-1/occludin	Mouse; human IEC lines/organoids	[8,35,36,45]
Goblet cell	Responder	STAT1/STAT3 supports MUC2 folding/secretion	Mouse; organoid	[46,47]
Paneth cell	Source + putative autocrine (mouse)	Subpopulation secretes IL-10 apically; possible autocrine circuit (mouse organoids, not shown in vivo/human)	Mouse organoids (proposed)	[15,48]
ILC2	Source	Inducible IL-10 production (IL-2/IL-4/IL-10/IL-27/NMU)	Mouse; human ILC2 in vitro	[14]
B cell (Breg)	Source	TLR2/MyD88/PI3K-dependent IL-10 production	Mouse; human B cells	[16]

NF-κB, nuclear factor kappa B; NLRP3, NOD-like receptor protein 3; IL-1β, interleukin-1 beta; IL-23, interleukin-23; IRF, interferon regulatory factor; CD86, cluster of differentiation 86; Foxp3, forkhead box P3; Th17, T helper 17 cells; RORγt, retinoic acid receptor-related orphan receptor gamma t; ER, endoplasmic reticulum; ZO-1, zonula occludens-1; IEC, intestinal epithelial cells; STAT1, signal transducer and activator of transcription 1; STAT3, signal transducer and activator of transcription 3; MUC2, mucin 2; ILC2, group 2 innate lymphoid cells; NMU, neuromedin U; TLR2, Toll-like receptor 2; MyD88, myeloid differentiation primary response 88; PI3K, phosphoinositide 3-kinase.

**Table 2:** Microbial signals that modulate the IL-10 axis.

Signal Type	Source	Target	Mechanism	Evidence Level	Refs.
Short-chain fatty acid	Butyrate	CD4^+^ T cell, IEC	HDAC inhibition + GPR43; PI3Kγ/phospho-mTOR; upregulates IL-10R1	Mouse; human in vitro	[69,70,71]
Tryptophan metabolite	Indole, IPA	IEC, immune cell	AHR activation	Mouse; in vitro	[75,76]
Bile acid derivative	GPBAR1 agonists	Macrophage	GPBAR1-CREB (mouse model); promotes IL-10 transcription, M2 polarization	Mouse	[78]
CpG DNA	Bacterial DNA (*C. sporogenes*)	B cell, DC	TLR9 activation; with butyrate induces IL-10^+^ Breg	Mouse; in vitro	[79]
TLR2 ligand	Lipoteichoic acid (*C. butyricum*, *L. johnsonii*)	Macrophage	TLR2/MyD88 or TLR1/2—STAT3	Mouse; in vitro	[80,82]
TLR2/6 ligand	*F. prausnitzii*	DC	TLR2/6-JNK; induces IL-10, IL-27, CD39; promotes Tr1	Human; in vitro	[83]
STING agonist	*Lacticaseibacillus rhamnosus* GG	Monocyte	STING/TBK1/NF-kB; induces IL-10	Mouse; in vitro	[84]
PSA	*B. fragilis*	CD4^+^ T cell	Requires IL-10^+^ T cells; suppresses IL-17	Mouse	[85]
Peptidoglycan	*B. adolescentis*	B cell	TLR2/ERK/p38; induces IL-10	Mouse; in vitro	[86]

IEC, intestinal epithelial cells; HDAC, histone deacetylase; GPR43, G protein-coupled receptor 43; PI3Kγ, phosphoinositide 3-kinase gamma; mTOR, mechanistic target of rapamycin; IPA, indole-3-propionic acid; AHR, aryl hydrocarbon receptor; GPBAR1, G protein-coupled bile acid receptor 1; CpG, cytosine-phosphate-guanine; CREB, cAMP response element-binding protein; TLR, Toll-like receptor; STAT3, signal transducer and activator of transcription 3; JNK, c-Jun N-terminal kinase; IL-27, interleukin-27; CD39, cluster of differentiation 39; Tr1, type 1 regulatory T cells; STING, stimulator of interferon genes; TBK1, TANK-binding kinase 1; NF-κB, nuclear factor kappa B; PSA, polysaccharide A; IL-17, interleukin-17; ERK, extracellular signal-regulated kinase; p38, p38 mitogen-activated protein kinase.

**Table 3:** Comparison of IL-10 targeted therapeutic strategies.

Platform	Representative Molecule	Development Stage (Verified)	Evidence Level	Refs
Systemic IL-10 supplementation	Recombinant human IL-10 (Tenovil)	Failed Phase II (CD)	Clinical (negative)	[119,121]
Gut-restricted delivery	AMT-101 (IL-10-cholix fusion)	Phase II completed (UC); LOMBARD and MARKET missed primary endpoint	Clinical (negative)	[123,124]
Local gene delivery	Lentiviral LV-IL-10	Preclinical (DSS colitis)	Preclinical	[128]
Engineered probiotic	*L. lactis* carrying IL-10 plasmid	Phase I completed (CD)	Early clinical	[129,130,131,147]
Conditionally activated prodrug	WTX-921 (IL-10 INDUKINE)	Preclinical (IBD models)	Preclinical	[133]
Engineered IL-10 variant	I87A/affinity-modified variants	Preclinical (structure-guided)	Preclinical	[24,134,135]
Antibody-IL-10 fusion	Anti-PD-1-IL-10 monomer	Phase I (solid tumors); FP008 NCT06990698 recruiting Phase 1	Early clinical	[116,137]
Cell therapy	Tr1 cells/IL-10^+^ Treg	Phase I/IIa (refractory CD); Ovasave Phase IIb terminated	Early clinical	[138,139]
Microbiome/metabolic	Butyrate, indole derivatives, MUFA supplementation, GPR120 agonist; enhances endogenous IL-10 production or signaling	Generally well-tolerated; modulates broader network; lack specificity; nonspecific immunosuppression	Early clinical (butyrate: randomized RCT reported as conference abstract, Crohn’s disease cohort; not an established Phase 2 UC program)	[71,146,148,149]

CD, Crohn’s disease; UC, ulcerative colitis; DSS, dextran sodium sulfate; IBD, inflammatory bowel disease; Tr1, type 1 regulatory T cells; Treg, regulatory T cells; MUFA, monounsaturated fatty acid; GPR120, G protein-coupled receptor 120; RCT, randomized controlled trial.

**Table 4:** Translational gap between animal models and human diseases.

Mechanistic Claim	Mouse/Preclinical Evidence	Human Evidence	Translational Gap	Refs.
IL-10 is non-redundant for intestinal homeostasis	IL-10^−/−^ and IL-10R^−/−^ mice develop spontaneous colitis dependent on resident bacteria; cell-specific models define macrophage/epithelial requirements	Inactivating *IL10/IL10R* mutations cause severe, early-onset, treatment-refractory IBD	Strong concordance on necessity; human mechanism less resolved	[3,4]
Macrophage autocrine IL-10 controls IL-23/commensal tolerance	Colonic macrophages engage autocrine IL-10 to limit IL-23 and maintain tolerance; metabolic reprogramming	Shp2 up-regulation disrupts IL-10—STAT3 signaling in IBD patient macrophage—analogous but not identical	Direct human autocrine IL-23 loop not established	[19,29,37]
Segment-specific (diet vs. microbiota) macrophage regulation	Small-intestinal IL-10^+^ macrophages diet-dependent; colonic macrophages commensal-dependent	Not directly shown in human intestine; inferred	Mouse-derived inference; human validation lacking	[18,19]
Paneth-cell IL-10 autocrine circuit	Subpopulation of IL-10^+^ Paneth cells in mouse small-intestinal organoids; ‘possible’ autocrine role	Not established in vivo or in humans	Major gap; model-derived hypothesis only	[15]
Microbiota-IL-10 reciprocity/dysbiosis	IL-10^−/−^ dysbiosis confounded by inflammation, antimicrobial peptides, genotype, housing, maternal transmission	Associational cohort data; correlation not causation	Causality unproven in humans	[91]
Therapeutic efficacy of IL-10 restoration	IL-10 or IL-10-gene delivery suppresses DSS/T cell-transfer colitis	First-generation rhIL-10 failed CD (no remission > placebo; dose-dependent anemia/thrombocytopenia); gut-restricted AMT-101 missed primary endpoints in UC (LOMBARD 17.1% vs. 20.0% placebo; MARKET missed)	Preclinical promise repeatedly fails to predict human efficacy	[122,124,125,126,128]

IL-10, interleukin-10; IL-10R, interleukin-10 receptor; IBD, inflammatory bowel disease; IL-23, interleukin-23; Shp2, SH2 domain-containing protein tyrosine phosphatase 2; STAT3, signal transducer and activator of transcription 3; DSS, dextran sodium sulfate; rhIL-10, recombinant human interleukin-10; CD, Crohn’s disease; UC, ulcerative colitis.

## Data Availability

No new data were generated or analyzed in this study.

## Abbreviations

The following abbreviations are used in this manuscript:

AHR	aryl hydrocarbon receptor
AMP	antimicrobial peptide
ATF6	activating transcription factor 6
ATG16L1	autophagy-related 16-like 1
Bhlhe40	basic helix-loop-helix family member e40
Blimp-1	B lymphocyte-induced maturation protein 1
Breg	regulatory B cell
CD	Crohn’s disease
c-FLIP	cellular FLICE-like inhibitory protein
c-MAF	musculoaponeurotic fibrosarcoma oncogene homolog
CRC	colorectal cancer
CREB	cAMP response element-binding protein
DCA	dichloroacetate
DDIT4	DNA damage-inducible transcript 4
DSS	dextran sodium sulfate
ER	endoplasmic reticulum
FcγR	Fc gamma receptor
Foxp3	forkhead box P3
GPR	G protein-coupled receptor
GPBAR1	G protein-coupled bile acid receptor 1
GRP78	glucose-regulated protein 78
HDAC	histone deacetylase
HSP47	heat shock protein 47
IBD	inflammatory bowel disease
ICOSL	inducible T cell costimulator ligand
IDO-1	indoleamine 2,3-dioxygenase 1
IEC	intestinal epithelial cell
IFN-γ	interferon gamma
IgG	immunoglobulin G
IL-10	interleukin-10
IL-10R	interleukin-10 receptor
ILC2	group 2 innate lymphoid cell
ILC3	group 3 innate lymphoid cell
IRI	ischemia–reperfusion injury
ISG15	interferon-stimulated gene 15
JAK1	Janus kinase 1
LPS	lipopolysaccharide
MAPK	mitogen-activated protein kinase
mTOR	mechanistic target of rapamycin
mTORC1	mechanistic target of rapamycin complex 1
MUFA	monounsaturated fatty acid
MUC2	mucin 2
NEC	necrotizing enterocolitis
NF-κB	nuclear factor kappa B
NLRP3	NOD-like receptor protein 3
NMU	neuromedin U
PGE_2_	prostaglandin E_2_
PI3K	phosphoinositide 3-kinase
PKM2	pyruvate kinase M2
PSA	polysaccharide A
REL	Rel proto-oncogene, NF-kB subunit
ROS	reactive oxygen species
SCFA	short-chain fatty acid
Shp2	SH2 domain-containing protein tyrosine phosphatase 2
STAT	signal transducer and activator of transcription
STING	stimulator of interferon genes
TBK1	TANK-binding kinase 1
TGF-β1	transforming growth factor beta 1
Th	T helper cells
TIL	tumor-infiltrating lymphocyte
TLR	Toll-like receptor
TNF-α	tumor necrosis factor alpha
Treg	regulatory T cell
TYK2	tyrosine kinase 2
UC	ulcerative colitis
VDR	vitamin D receptor
VLC	very-long-chain
WISP-1	WNT1-inducible signaling pathway protein 1
ZO-1	zonula occludens-1
βTrCP	beta-transducin repeat-containing protein

## References

[1] Yang X., Guo H., Zou M. (2026). Inflammatory bowel diseases: Pathological mechanisms and therapeutic perspectives. Mol. Biomed..

[2] Sabat R., Grütz G., Warszawska K., Kirsch S., Witte E., Wolk K., Geginat J. (2010). Biology of interleukin-10. Cytokine Growth Factor Rev..

[3] Kotlarz D., Beier R., Murugan D., Diestelhorst J., Jensen O., Boztug K., Pfeifer D., Kreipe H., Pfister E.D., Baumann U. (2012). Loss of Interleukin-10 Signaling and Infantile Inflammatory Bowel Disease: Implications for Diagnosis and Therapy. Gastroenterology.

[4] Shouval D.S., Ouahed J., Biswas A., Goettel J.A., Horwitz B.H., Klein C., Muise A.M., Snapper S.B. (2014). Interleukin 10 receptor signaling: Master regulator of intestinal mucosal homeostasis in mice and humans. Adv. Immunol..

[5] York A.G., Skadow M.H., Oh J., Qu R., Zhou Q.D., Hsieh W.-Y., Mowel W.K., Brewer J.R., Kaffe E., Williams K.J. (2024). IL-10 constrains sphingolipid metabolism to limit inflammation. Nature.

[6] Liu H., Zhao X., Yu T., Yu Y., Yao S., Yang W., Cong Y. (2025). Metabolic regulation of T cell production of IL-10 and IL-22 protects against intestinal inflammation. Precis. Clin. Med..

[7] Landuyt A.E., Klocke B.J., Duck L.W., Kemp K.M., Muir R.Q., Jennings M.S., Blum S.I., Tse H.M., Lee G., Morrow C.D. (2021). ICOS ligand and IL-10 synergize to promote host–microbiota mutualism. Proc. Natl. Acad. Sci. USA.

[8] Quiros M., Nishio H., Neumann P.A., Siuda D., Brazil J.C., Azcutia V., Hilgarth R., O’Leary M.N., Garcia-Hernandez V., Leoni G. (2017). Macrophage-derived IL-10 mediates mucosal repair by epithelial WISP-1 signaling. J. Clin. Investig..

[9] Steen E.H., Wang X., Balaji S., Butte M.J., Bollyky P.L., Keswani S.G. (2020). The Role of the Anti-Inflammatory Cytokine Interleukin-10 in Tissue Fibrosis. Adv. Wound Care.

[10] Oft M. (2019). Immune regulation and cytotoxic T cell activation of IL-10 agonists–Preclinical and clinical experience. Semin. Immunol..

[11] Zigmond E., Bernshtein B., Friedlander G., Walker C.R., Yona S., Kim K.-W., Brenner O., Krauthgamer R., Varol C., Müller W. (2014). Macrophage-Restricted Interleukin-10 Receptor Deficiency, but Not IL-10 Deficiency, Causes Severe Spontaneous Colitis. Immunity.

[12] Morhardt T.L., Hayashi A., Ochi T., Quirós M., Kitamoto S., Nagao-Kitamoto H., Kuffa P., Atarashi K., Honda K., Kao J.Y. (2019). IL-10 produced by macrophages regulates epithelial integrity in the small intestine. Sci. Rep..

[13] Girard-Madoux M.J.H., Ober-Blöbaum J.L., Costes L.M.M., Kel J.M., Lindenbergh-Kortleve D.J., Brouwers-Haspels I., Heikema A.P., Samsom J.N., Clausen B.E. (2016). IL-10 control of CD11c+ myeloid cells is essential to maintain immune homeostasis in the small and large intestine. Oncotarget.

[14] Bando J.K., Gilfillan S., Di Luccia B., Fachi J.L., Sécca C., Cella M., Colonna M. (2020). ILC2s are the predominant source of intestinal ILC-derived IL-10. J. Exp. Med..

[15] Nguyen H., Di Cara F., Wang J., Stadnyk A.W. (2025). Paneth Cells Are a Constitutive Source of IL-10 in Mouse Small Intestinal Organoids. Organoids.

[16] Mishima Y., Oka A., Liu B., Herzog J.W., Eun C.S., Fan T.-J., Bulik-Sullivan E., Carroll I.M., Hansen J.J., Chen L. (2019). Microbiota maintain colonic homeostasis by activating TLR2/MyD88/PI3K signaling in IL-10–producing regulatory B cells. J. Clin. Investig..

[17] Roers A., Siewe L., Strittmatter E., Deckert M., Schlüter D., Stenzel W., Gruber A.D., Krieg T., Rajewsky K., Müller W. (2004). T Cell–specific Inactivation of the Interleukin 10 Gene in Mice Results in Enhanced T Cell Responses but Normal Innate Responses to Lipopolysaccharide or Skin Irritation. J. Exp. Med..

[18] Ochi T., Feng Y., Kitamoto S., Nagao-Kitamoto H., Kuffa P., Atarashi K., Honda K., Teitelbaum D.H., Kamada N. (2016). Diet-dependent, microbiota-independent regulation of IL-10-producing lamina propria macrophages in the small intestine. Sci. Rep..

[19] Ueda Y., Kayama H., Jeon S.G., Kusu T., Isaka Y., Rakugi H., Yamamoto M., Takeda K. (2010). Commensal microbiota induce LPS hyporesponsiveness in colonic macrophages via the production of IL-10. Int. Immunol..

[20] Brockmann L., Tran A., Huang Y., Edwards M., Ronda C., Wang H.H., Ivanov I.I. (2023). Intestinal microbiota-specific Th17 cells possess regulatory properties and suppress effector T cells via c-MAF and IL-10. Immunity.

[21] Kotenko S.V., Krause C.D., Izotova L.S., Pollack B.P., Wu W., Pestka S. (1997). Identification and functional characterization of a second chain of the interleukin-10 receptor complex. EMBO J..

[22] Spencer S.D., Di Marco F., Hooley J., Pitts-Meek S., Bauer M., Ryan A.M., Sordat B., Gibbs V.C., Aguet M. (1998). The Orphan Receptor CRF2-4 Is an Essential Subunit of the Interleukin 10 Receptor. J. Exp. Med..

[23] Yoon S.I., Logsdon N.J., Sheikh F., Donnelly R.P., Walter M.R. (2006). Conformational Changes Mediate Interleukin-10 Receptor 2 (IL-10R2) Binding to IL-10 and Assembly of the Signaling Complex. J. Biol. Chem..

[24] Saxton R.A., Tsutsumi N., Su L.L., Abhiraman G.C., Mohan K., Henneberg L.T., Aduri N.G., Gati C., Garcia K.C. (2021). Structure-based decoupling of the pro- and anti-inflammatory functions of interleukin-10. Science.

[25] Kominsky D.J., Campbell E.L., Ehrentraut S.F., Wilson K.E., Kelly C.J., Glover L.E., Collins C.B., Bayless A.J., Saeedi B., Dobrinskikh E. (2014). IFN-γ–Mediated Induction of an Apical IL-10 Receptor on Polarized Intestinal Epithelia. J. Immunol..

[26] Wei S.H.Y., Ming-Lum A., Liu Y., Wallach D., Ong C.J., Chung S.W., Moore K.W., Mui A.L.F. (2006). Proteasome-Mediated Proteolysis of the Interleukin-10 Receptor Is Important for Signal Downregulation. J. Interferon Cytokine Res..

[27] Buday L., Jiang H., Lu Y., Yuan L., Liu J. (2011). Regulation of Interleukin-10 Receptor Ubiquitination and Stability by Beta-TrCP-Containing Ubiquitin E3 Ligase. PLoS ONE.

[28] Hutchins A.P., Takahashi Y., Miranda-Saavedra D. (2015). Genomic analysis of LPS-stimulated myeloid cells identifies a common pro-inflammatory response but divergent IL-10 anti-inflammatory responses. Sci. Rep..

[29] Krause P., Morris V., Greenbaum J.A., Park Y., Bjoerheden U., Mikulski Z., Muffley T., Shui J.-W., Kim G., Cheroutre H. (2015). IL-10-producing intestinal macrophages prevent excessive antibacterial innate immunity by limiting IL-23 synthesis. Nat. Commun..

[30] Shouval D.S., Biswas A., Kang Y.H., Griffith A.E., Konnikova L., Mascanfroni I.D., Redhu N.S., Frei S.M., Field M., Doty A.L. (2016). Interleukin 1β Mediates Intestinal Inflammation in Mice and Patients With Interleukin 10 Receptor Deficiency. Gastroenterology.

[31] Huber S., Gagliani N., Esplugues E., O’Connor W., Huber F.J., Chaudhry A., Kamanaka M., Kobayashi Y., Booth C.J., Rudensky A.Y. (2011). Th17 Cells Express Interleukin-10 Receptor and Are Controlled by Foxp3− and Foxp3+ Regulatory CD4+ T Cells in an Interleukin-10-Dependent Manner. Immunity.

[32] Hsu P., Santner-Nanan B., Hu M., Skarratt K., Lee C.H., Stormon M., Wong M., Fuller S.J., Nanan R. (2015). IL-10 Potentiates Differentiation of Human Induced Regulatory T Cells via STAT3 and Foxo1. J. Immunol..

[33] Chaudhry A., Samstein R.M., Treuting P., Liang Y., Pils M.C., Heinrich J.-M., Jack R.S., Wunderlich F.T., Brüning J.C., Müller W. (2011). Interleukin-10 Signaling in Regulatory T Cells Is Required for Suppression of Th17 Cell-Mediated Inflammation. Immunity.

[34] Shouval D.S., Konnikova L., Griffith A.E., Wall S.M., Biswas A., Werner L., Nunberg M., Kammermeier J., Goettel J.A., Anand R. (2017). Enhanced TH17 Responses in Patients with IL10 Receptor Deficiency and Infantile-onset IBD. Inflamm. Bowel Dis..

[35] Bharhani M.S., Borojevic R., Basak S., Ho E., Zhou P., Croitoru K. (2006). IL-10 protects mouse intestinal epithelial cells from Fas-induced apoptosis via modulating Fas expression and altering caspase-8 and FLIP expression. Am. J. Physiol. Gastrointest. Liver Physiol..

[36] Sun X., Yang H., Nose K., Nose S., Haxhija E.Q., Koga H., Feng Y., Teitelbaum D.H. (2008). Decline in intestinal mucosal IL-10 expression and decreased intestinal barrier function in a mouse model of total parenteral nutrition. Am. J. Physiol. Gastrointest. Liver Physiol..

[37] Xiao P., Zhang H., Zhang Y., Zheng M., Liu R., Zhao Y., Zhang X., Cheng H., Cao Q., Ke Y. (2019). Phosphatase Shp2 exacerbates intestinal inflammation by disrupting macrophage responsiveness to interleukin-10. J. Exp. Med..

[38] Aschenbrenner D., Quaranta M., Banerjee S., Ilott N., Jansen J., Steere B., Chen Y.-H., Ho S., Cox K., Arancibia-Cárcamo C.V. (2021). Deconvolution of monocyte responses in inflammatory bowel disease reveals an IL-1 cytokine network that regulates IL-23 in genetic and acquired IL-10 resistance. Gut.

[39] Melgar S., Yeung M.M.W., Bas A., Forsberg G., Suhr O., ÖBerg Å., Hammarström S., Danielsson Å., Hammarström M.L. (2003). Over-expression of interleukin 10 in mucosal T cells of patients with active ulcerative colitis. Clin. Exp. Immunol..

[40] Pavel C., Diculescu M.M., Ilie M., Plotogea O.-M., Sandru V., Enache V., Gheonea D.-I., Jichitu A., Constantinescu A., Serban R.-E. (2025). Immunohistochemistry Analysis in Inflammatory Bowel Disease—Should We Bring to Light Interleukin-10?. Biomedicines.

[41] Waidmann M., Allemand Y., Lehmann J., di Genaro S., Bücheler N., Hamann A., Autenrieth I.B. (2002). Microflora reactive IL-10 producing regulatory T cells are present in the colon of IL-2 deficient mice but lack efficacious inhibition of IFN-γ and TNF-α production. Gut.

[42] Ip W.K.E., Hoshi N., Shouval D.S., Snapper S., Medzhitov R. (2017). Anti-inflammatory effect of IL-10 mediated by metabolic reprogramming of macrophages. Science.

[43] Buelens C., Willems F., Delvaux A., Piérard G., Delville J.P., Velu T., Goldman M. (1995). Interleukin-10 differentially regulates B7-1 (CD80) and B7-2 (CD86) expression on human peripheral blood dendritic cells. Eur. J. Immunol..

[44] Murai M., Turovskaya O., Kim G., Madan R., Karp C.L., Cheroutre H., Kronenberg M. (2009). Interleukin 10 acts on regulatory T cells to maintain expression of the transcription factor Foxp3 and suppressive function in mice with colitis. Nat. Immunol..

[45] Shkoda A., Ruiz P.A., Daniel H., Kim S.C., Rogler G., Sartor R.B., Haller D. (2007). Interleukin-10 Blocked Endoplasmic Reticulum Stress in Intestinal Epithelial Cells: Impact on Chronic Inflammation. Gastroenterology.

[46] Hasnain S.Z., Tauro S., Das I., Tong H., Chen A.C.H., Jeffery P.L., McDonald V., Florin T.H., McGuckin M.A. (2013). IL-10 Promotes Production of Intestinal Mucus by Suppressing Protein Misfolding and Endoplasmic Reticulum Stress in Goblet Cells. Gastroenterology.

[47] Schwerbrock N.M.J., Makkink M.K., van der Sluis M., Büller H.A., Einerhand A.W.C., Sartor R.B., Dekker J. (2004). Interleukin 10-Deficient Mice Exhibit Defective Colonic Muc2 Synthesis Before and After Induction of Colitis by Commensal Bacteria. Inflamm. Bowel Dis..

[48] Chamaillard M., Berkowitz L., Pardo-Roa C., Ramírez G., Vallejos O.P., Sebastián V.P., Riedel C.A., Álvarez-Lobos M., Bueno S.M. (2019). The absence of interleukin 10 affects the morphology, differentiation, granule content and the production of cryptidin-4 in Paneth cells in mice. PLoS ONE.

[49] Ogger P.P., Murray P.J. (2025). Dissecting inflammation in the immunemetabolomic era. Cell. Mol. Life Sci..

[50] Nguyen H.D., Aljamaei H.M., Stadnyk A.W. (2021). The Production and Function of Endogenous Interleukin-10 in Intestinal Epithelial Cells and Gut Homeostasis. Cell. Mol. Gastroenterol. Hepatol..

[51] Saraiva M., Vieira P., O’Garra A. (2019). Biology and therapeutic potential of interleukin-10. J. Exp. Med..

[52] Simon J.M., Davis J.P., Lee S.E., Schaner M.R., Gipson G.R., Weiser M., Sartor R.B., Herfarth H.H., Rahbar R., Sadiq T.S. (2016). Alterations to chromatin in intestinal macrophages link IL-10 deficiency to inappropriate inflammatory responses. Eur. J. Immunol..

[53] Stoolman J.S., Grant R.A., Billingham L.K., Poor T.A., Weinberg S.E., Harding M.C., Lu Z., Miska J., Szibor M., Budinger G.R.S. (2025). Mitochondria complex III–generated superoxide is essential for IL-10 secretion in macrophages. Sci. Adv..

[54] Toller-Kawahisa J.E., Viacava P.R., Palsson-McDermott E.M., Nascimento D.C., Cervantes-Silva M.P., O’Carroll S.M., Zotta A., Damasceno L.E.A., Públio G.A., Forti P. (2025). Metabolic reprogramming of macrophages by PKM2 promotes IL-10 production via adenosine. Cell Rep..

[55] Koelink P.J., Bloemendaal F.M., Li B., Westera L., Vogels E.W.M., van Roest M., Gloudemans A.K., van ‘t Wout A.B., Korf H., Vermeire S. (2020). Anti-TNF therapy in IBD exerts its therapeutic effect through macrophage IL-10 signalling. Gut.

[56] Kühn R., Löhler J., Rennick D., Rajewsky K., Müller W. (1993). Interleukin-10-deficient mice develop chronic enterocolitis. Cell.

[57] Jarry A., Bossard C., Bou-Hanna C., Masson D., Espaze E., Denis M.G., Laboisse C.L. (2008). Mucosal IL-10 and TGF-β play crucial roles in preventing LPS-driven, IFN-γ–mediated epithelial damage in human colon explants. J. Clin. Investig..

[58] Pan D., Das A., Lala W., Kenway-Lynch C.S., Liu D.X., Veazey R.S., Pahar B. (2013). Interleukin-10 prevents epithelial cell apoptosis by regulating IFNγ and TNFα expression in rhesus macaque colon explants. Cytokine.

[59] He Z., Feng Y.-d., Zhang Y.-x., Gao X., Liu J.-j., Liu S., Wu G.-q. (2025). IL-10 alleviates ulcerative colitis by regulating mitochondrial function through reducing ISG15 expression. Cell. Signal..

[60] Serramito-Gómez I., Boada-Romero E., Villamuera R., Fernández-Cabrera Á., Cedillo J.L., Martín-Regalado Á., Carding S., Mayer U., Powell P.P., Wileman T. (2020). Regulation of cytokine signaling through direct interaction between cytokine receptors and the ATG16L1 WD40 domain. Nat. Commun..

[61] Serramito-Gómez I., Terraza-Silvestre E., Fernández-Cabrera Á., Villamuera R., Pimentel-Muiños F.X. (2022). ATG16L1 WD40 domain-dependent IL10R (interleukin 10 receptor) signaling is insensitive to the T300A Crohn disease risk polymorphism. Autophagy.

[62] Schneider-Stock R., Lorén V., Cabré E., Ojanguren I., Domènech E., Pedrosa E., García-Jaraquemada A., Mañosa M., Manyé J. (2015). Interleukin-10 Enhances the Intestinal Epithelial Barrier in the Presence of Corticosteroids through p38 MAPK Activity in Caco-2 Monolayers: A Possible Mechanism for Steroid Responsiveness in Ulcerative Colitis. PLoS ONE.

[63] Poholek A.C., Jankovic D., Villarino A.V., Petermann F., Hettinga A., Shouval D.S., Snapper S.B., Kaech S.M., Brooks S.R., Vahedi G. (2016). IL-10 induces a STAT3-dependent autoregulatory loop in TH2 cells that promotes Blimp-1 restriction of cell expansion via antagonism of STAT5 target genes. Sci. Immunol..

[64] Ito T., Li B., Sakaguchi T., Yagita-Sakamaki M., Itoi H., Murakami M., Wu R., Fukada A., Motooka D., Ogino T. (2026). The interleukin-10/interleukin-10Rα axis in fibroblasts limits large intestinal pathology by suppressing Type I interferon signaling. Int. Immunol..

[65] Zou Y., Zhang J., Mao W., Jiang S., Xu C., Meng J., Dong H., Mou Y. (2026). Interleukin-10 Derived Apoptotic Vesicles Enhance Scarless Skin Healing by Modulating Fibroblast Metabolism and Fibrosis Pathways. Cell Prolif..

[66] Li J., Jacobse J., Pilat J.M., Kaur H., Gu W., Kang S.W., Rusznak M., Huang H.-I., Barrera J., Oloo P.A. (2025). Interleukin-10 production by innate lymphoid cells restricts intestinal inflammation in mice. Mucosal Immunol..

[67] Howard E., Lewis G., Galle-Treger L., Hurrell B.P., Helou D.G., Shafiei-Jahani P., Painter J.D., Muench G.A., Soroosh P., Akbari O. (2021). IL-10 production by ILC2s requires Blimp-1 and cMaf, modulates cellular metabolism, and ameliorates airway hyperreactivity. J. Allergy Clin. Immunol..

[68] Liu Q., Wang Y., Li Y., Wang Y., Liu W., Rosen S., Liang F., Xie T., Xia Y. (2026). Calcitriol–PKM2 axis drives transcriptional and metabolic reprogramming of ILCPs into intestinal dual-cytokine-producing ILC3s. Proc. Natl. Acad. Sci. USA.

[69] Yang W., Yu T., Liu X., Yao S., Khanipov K., Golovko G., Olivares-Villagómez D., Cong Y. (2024). Microbial metabolite butyrate modulates granzyme B in tolerogenic IL-10 producing Th1 cells to regulate intestinal inflammation. Gut Microbes.

[70] Wen S.-H., Zhang Y.-N., Shen J.-T., Guo Y., Chang Z.-N., Zhang H.-F., Liu Z.-M., Zhang X.-Y. (2026). PD-1 blockade promotes mucosal CD4+ T cell IL-10 production through altering microbiota to reduce intestinal ischemia reperfusion injury. Gut Microbes.

[71] Zheng L., Kelly C.J., Battista K.D., Schaefer R., Lanis J.M., Alexeev E.E., Wang R.X., Onyiah J.C., Kominsky D.J., Colgan S.P. (2017). Microbial-Derived Butyrate Promotes Epithelial Barrier Function through IL-10 Receptor-Dependent Repression of Claudin-2. J. Immunol..

[72] Sun M., Wu W., Chen L., Yang W., Huang X., Ma C., Chen F., Xiao Y., Zhao Y., Ma C. (2018). Microbiota-derived short-chain fatty acids promote Th1 cell IL-10 production to maintain intestinal homeostasis. Nat. Commun..

[73] Luu M., Pautz S., Kohl V., Singh R., Romero R., Lucas S., Hofmann J., Raifer H., Vachharajani N., Carrascosa L.C. (2019). The short-chain fatty acid pentanoate suppresses autoimmunity by modulating the metabolic-epigenetic crosstalk in lymphocytes. Nat. Commun..

[74] Yu T., Yang W., Bilotta A.J., Yao S., Lee J.-H., Zhao Q., Zhou J., Ordog T., Dulai P.S., Cong Y. (2026). Butyrate epigenetically licenses sustained epithelial-T cell crosstalk for intestinal immune tolerance. Nat. Commun..

[75] Powell D.N., Swimm A., Sonowal R., Bretin A., Gewirtz A.T., Jones R.M., Kalman D. (2020). Indoles from the commensal microbiota act via the AHR and IL-10 to tune the cellular composition of the colonic epithelium during aging. Proc. Natl. Acad. Sci. USA.

[76] Alexeev E.E., Lanis J.M., Kao D.J., Campbell E.L., Kelly C.J., Battista K.D., Gerich M.E., Jenkins B.R., Walk S.T., Kominsky D.J. (2018). Microbiota-Derived Indole Metabolites Promote Human and Murine Intestinal Homeostasis through Regulation of Interleukin-10 Receptor. Am. J. Pathol..

[77] Ming D., Xu X., Jiang X., Li Y., Sun W., Xiang J., Huang M., Pi Y., Li X. (2024). Indole-3-propionic acid enhances growth performance and reduces diarrhea via modulating redox status and intestinal inflammation in weaned piglets. Anim. Nutr..

[78] Biagioli M., Carino A., Cipriani S., Francisci D., Marchianò S., Scarpelli P., Sorcini D., Zampella A., Fiorucci S. (2017). The Bile Acid Receptor GPBAR1 Regulates the M1/M2 Phenotype of Intestinal Macrophages and Activation of GPBAR1 Rescues Mice from Murine Colitis. J. Immunol..

[79] Luu M., Krause F.F., Monning H., Wempe A., Leister H., Mainieri L., Staudt S., Ziegler-Martin K., Mangold K., Kappelhoff N. (2025). Dissecting the metabolic signaling pathways by which microbial molecules drive the differentiation of regulatory B cells. Mucosal Immunol..

[80] Hayashi A., Sato T., Kamada N., Mikami Y., Matsuoka K., Hisamatsu T., Hibi T., Roers A., Yagita H., Ohteki T. (2013). A Single Strain of Clostridium butyricum Induces Intestinal IL-10-Producing Macrophages to Suppress Acute Experimental Colitis in Mice. Cell Host Microbe.

[81] Goodman A.L., Jeon S.G., Kayama H., Ueda Y., Takahashi T., Asahara T., Tsuji H., Tsuji N.M., Kiyono H., Ma J.S. (2012). Probiotic Bifidobacterium breve Induces IL-10-Producing Tr1 Cells in the Colon. PLoS Pathog..

[82] Jia D.-J.-C., Wang Q.-W., Hu Y.-Y., He J.-M., Ge Q.-W., Qi Y.-D., Chen L.-Y., Zhang Y., Fan L.-N., Lin Y.-F. (2022). Lactobacillus johnsonii alleviates colitis by TLR1/2-STAT3 mediated CD206+ macrophages IL-10 activation. Gut Microbes.

[83] Alameddine J., Godefroy E., Papargyris L., Sarrabayrouse G., Tabiasco J., Bridonneau C., Yazdanbakhsh K., Sokol H., Altare F., Jotereau F. (2019). Faecalibacterium prausnitzii Skews Human DC to Prime IL10-Producing T Cells Through TLR2/6/JNK Signaling and IL-10, IL-27, CD39, and IDO-1 Induction. Front. Immunol..

[84] Si W., Zhao X., Li R., Li Y., Ma C., Zhao X., Bugno J., Qin Y., Zhang J., Liu H. (2025). Lactobacillus rhamnosus GG induces STING-dependent IL-10 in intestinal monocytes and alleviates inflammatory colitis in mice. J. Clin. Investig..

[85] Mazmanian S.K., Round J.L., Kasper D.L. (2008). A microbial symbiosis factor prevents intestinal inflammatory disease. Nature.

[86] Lee S., Lee Y., Lee H.-S., Yu J., Kim K., Kim T.-Y., Lee S.-H., Qiao Y., Kim S., Kweon M.-N. (2026). Peptidoglycan from Bifidobacterium adolescentis enhances IL-10 production in regulatory B cells to alleviate gut inflammation. Gut Microbes.

[87] Maharshak N., Packey C.D., Ellermann M., Manick S., Siddle J.P., Huh E.Y., Plevy S., Sartor R.B., Carroll I.M. (2014). Altered enteric microbiota ecology in interleukin 10-deficient mice during development and progression of intestinal inflammation. Gut Microbes.

[88] Miyoshi J., Lee S.T.M., Kennedy M.S., Puertolas M.V., Frith M.E., Koval J.C., Miyoshi S., Antonopoulos D.A., Leone V.A., Chang E.B. (2020). Metagenomic Alterations in Gut Microbiota Precede and Predict Onset of Colitis in the IL10 Gene-Deficient Murine Model. Cell. Mol. Gastroenterol. Hepatol..

[89] Gunasekera D.C., Ma J., Vacharathit V., Shah P., Ramakrishnan A., Uprety P., Shen Z., Sheh A., Brayton C.F., Whary M.T. (2020). The development of colitis in Il10 mice is dependent on IL-22. Mucosal Immunol..

[90] Harris N., Duque-Correa M.A., Karp N.A., McCarthy C., Forman S., Goulding D., Sankaranarayanan G., Jenkins T.P., Reid A.J., Cambridge E.L. (2019). Exclusive dependence of IL-10Rα signalling on intestinal microbiota homeostasis and control of whipworm infection. PLoS Pathog..

[91] Schulfer A.F., Battaglia T., Alvarez Y., Bijnens L., Ruiz V.E., Ho M., Robinson S., Ward T., Cox L.M., Rogers A.B. (2017). Intergenerational transfer of antibiotic-perturbed microbiota enhances colitis in susceptible mice. Nat. Microbiol..

[92] He Z., Xie H., Xu H., Wu J., Zeng W., He Q., Jobin C., Jin S., Lan P. (2024). Chemotherapy-induced microbiota exacerbates the toxicity of chemotherapy through the suppression of interleukin-10 from macrophages. Gut Microbes.

[93] Fung T.C., Bessman N.J., Hepworth M.R., Kumar N., Shibata N., Kobuley D., Wang K., Ziegler C.G.K., Goc J., Shima T. (2016). Lymphoid-Tissue-Resident Commensal Bacteria Promote Members of the IL-10 Cytokine Family to Establish Mutualism. Immunity.

[94] Vidal V.M., Montes-Cobos E., Canto F.B., Bozza M.T. (2026). The different meanings of tolerating the gut microbiome. mBio.

[95] Franke A., Balschun T., Karlsen T.H., Sventoraityte J., Nikolaus S., Mayr G., Domingues F.S., Albrecht M., Nothnagel M., Ellinghaus D. (2008). Sequence variants in IL10, ARPC2 and multiple other loci contribute to ulcerative colitis susceptibility. Nat. Genet..

[96] Griffin H., Ceron-Gutierrez L., Gharahdaghi N., Ebrahimi S., Davies S., Loo P.S., Szabo A., Williams E., Mukhopadhyay A., McLoughlin L. (2024). Neutralizing Autoantibodies against Interleukin-10 in Inflammatory Bowel Disease. N. Engl. J. Med..

[97] Shouval D.S., Biswas A., Goettel J.A., McCann K., Conaway E., Redhu N.S., Mascanfroni I.D., Al Adham Z., Lavoie S., Ibourk M. (2014). Interleukin-10 Receptor Signaling in Innate Immune Cells Regulates Mucosal Immune Tolerance and Anti-Inflammatory Macrophage Function. Immunity.

[98] Emami C.N., Chokshi N., Wang J., Hunter C., Guner Y., Goth K., Wang L., Grishin A., Ford H.R. (2012). Role of interleukin-10 in the pathogenesis of necrotizing enterocolitis. Am. J. Surg..

[99] Dvorak B., Halpern M.D., Holubec H., Dvorakova K., Dominguez J.A., Williams C.S., Meza Y.G., Kozakova H., McCuskey R.S. (2003). Maternal Milk Reduces Severity of Necrotizing Enterocolitis and Increases Intestinal IL-10 in a Neonatal Rat Model. Pediatr. Res..

[100] Ahlers J., Mantei A., Lozza L., Stäber M., Heinrich F., Bacher P., Hohnstein T., Menzel L., Yüz S.G., Alvarez-Simon D. (2022). A Notch/STAT3-driven Blimp-1/c-Maf-dependent molecular switch induces IL-10 expression in human CD4+ T cells and is defective in Crohn’s disease patients. Mucosal Immunol..

[101] Uchida A.M., Boden E.K., James E.A., Shows D.M., Konecny A.J., Lord J.D. (2020). Escherichia coli–Specific CD4+ T Cells Have Public T-Cell Receptors and Low Interleukin 10 Production in Crohn’s Disease. Cell. Mol. Gastroenterol. Hepatol..

[102] Brockmann L., Soukou S., Steglich B., Czarnewski P., Zhao L., Wende S., Bedke T., Ergen C., Manthey C., Agalioti T. (2018). Molecular and functional heterogeneity of IL-10-producing CD4+ T cells. Nat. Commun..

[103] Zingarelli B., Yang Z., Hake P.W., Denenberg A., Wong H.R. (2001). Absence of endogenous interleukin 10 enhances early stress response during post-ischaemic injury in mice intestine. Gut.

[104] Stoffels B., Schmidt J., Nakao A., Nazir A., Chanthaphavong R.S., Bauer A.J. (2009). Role of interleukin 10 in murine postoperative ileus. Gut.

[105] Stein K., Lysson M., Schumak B., Vilz T., Specht S., Heesemann J., Roers A., Kalff J.C., Wehner S. (2018). Leukocyte-Derived Interleukin-10 Aggravates Postoperative Ileus. Front. Immunol..

[106] Schopf L.R., Hoffmann K.F., Cheever A.W., Urban J.F., Wynn T.A. (2002). IL-10 Is Critical for Host Resistance and Survival During Gastrointestinal Helminth Infection. J. Immunol..

[107] Webster H.C., Gamino V., Andrusaite A.T., Ridgewell O.J., McCowan J., Shergold A.L., Heieis G.A., Milling S.W.F., Maizels R.M., Perona-Wright G. (2022). Tissue-based IL-10 signalling in helminth infection limits IFNγ expression and promotes the intestinal Th2 response. Mucosal Immunol..

[108] Dann S.M., Le C., Choudhury B.K., Liu H., Saldarriaga O., Hanson E.M., Cong Y., Eckmann L., Flynn J.L. (2014). Attenuation of Intestinal Inflammation in Interleukin-10-Deficient Mice Infected with Citrobacter rodentium. Infect. Immun..

[109] Mukhopadhyay S., Heinz E., Porreca I., Alasoo K., Yeung A., Yang H.-T., Schwerd T., Forbester J.L., Hale C., Agu C.A. (2020). Loss of IL-10 signaling in macrophages limits bacterial killing driven by prostaglandin E2. J. Exp. Med..

[110] Kitamura H., Yamamoto S., Nakase H., Matsuura M., Honzawa Y., Matsumura K., Takeda Y., Uza N., Nagata K., Chiba T. (2011). Role of heat shock protein 47 in intestinal fibrosis of experimental colitis. Biochem. Biophys. Res. Commun..

[111] Borowiec A.M., Sydora B.C., Doyle J., Guan L.L., Churchill T.A., Madsen K., Fedorak R.N. (2012). Small bowel fibrosis and systemic inflammatory response after ileocolonic anastomosis in IL-10 null mice. J. Surg. Res..

[112] Kitani A., Fuss I., Nakamura K., Kumaki F., Usui T., Strober W. (2003). Transforming Growth Factor (TGF)-β1–producing Regulatory T Cells Induce Smad-mediated Interleukin 10 Secretion That Facilitates Coordinated Immunoregulatory Activity and Amelioration of TGF-β1–mediated Fibrosis. J. Exp. Med..

[113] Hecht J.R., Lonardi S., Bendell J., Sim H.W., Macarulla T., Lopez C.D., Van Cutsem E., Muñoz Martin A.J., Park J.O., Greil R. (2021). Randomized Phase III Study of FOLFOX Alone or With Pegilodecakin as Second-Line Therapy in Patients With Metastatic Pancreatic Cancer That Progressed After Gemcitabine (SEQUOIA). J. Clin. Oncol..

[114] Gu T., De Jesus M., Gallagher H.C., Burris T.P., Egilmez N.K. (2017). Oral IL-10 suppresses colon carcinogenesis via elimination of pathogenicCD4(+) T-cells and induction of antitumor CD8(+) T-cell activity. Oncoimmunology.

[115] Shiri A.M., Fard-Aghaie M., Bedke T., Papazoglou E.D., Sabihi M., Zazara D.E., Zhang S., Lücke J., Seeger P., Evers M. (2024). Foxp3 + Treg-derived IL-10 promotes colorectal cancer-derived lung metastasis. Sci. Rep..

[116] Gu C., Guo J., Zhou C., Hu P., Wu X., Ding J., Zhou X., Zeng L., Yu W., Ou Y. (2026). An innovative engineered IL-10 monomer strengthens T cell-mediated anti-tumor responses through anti-PD-1 cis-delivery. Cell Rep. Med..

[117] Guo Y., Xie Y.-Q., Gao M., Zhao Y., Franco F., Wenes M., Siddiqui I., Bevilacqua A., Wang H., Yang H. (2021). Metabolic reprogramming of terminally exhausted CD8+ T cells by IL-10 enhances anti-tumor immunity. Nat. Immunol..

[118] Gruber S.G., Gloria Luciani M., Grundtner P., Zdanov A., Gasche C. (2008). Differential signaling of cmvIL-10 through common variants of the IL-10 receptor 1. Eur. J. Immunol..

[119] Buruiana F.E., Solà I., Alonso-Coello P. (2010). Recombinant human interleukin 10 for induction of remission in Crohn’s disease. Cochrane Database Syst. Rev..

[120] Marlow G.J. (2013). Why interleukin-10 supplementation does not work in Crohn’s disease patients. World J. Gastroenterol..

[121] Schreiber S., Fedorak R.N., Nielsen O.H., Wild G., Williams C.N., Nikolaus S., Jacyna M., Lashner B.A., Gangl A., Rutgeerts P. (2000). Safety and efficacy of recombinant human interleukin 10 in chronic active Crohn’s disease. Gastroenterology.

[122] Li B., Gurung P., Malireddi R.K.S., Vogel P., Kanneganti T.-D., Geiger T.L. (2015). IL-10 engages macrophages to shift Th17 cytokine dependency and pathogenicity during T-cell-mediated colitis. Nat. Commun..

[123] Fay N.C., Muthusamy B.-P., Nyugen L.P., Desai R.C., Taverner A., MacKay J., Seung M., Hunter T., Liu K., Chandalia A. (2020). A Novel Fusion of IL-10 Engineered to Traffic across Intestinal Epithelium to Treat Colitis. J. Immunol..

[124] Posch M., Tanase R., Maisaia K., Jucov A., Geier C., Hotson D., Hamdy O., Whitney J.A., Garovoy M., Kanwar B. (2021). P294 AMT-101, a gut selective oral IL-10 fusion: Results from a Phase 1b study in patients with active Ulcerative Colitis. J. Crohn’s Colitis.

[125] (2020). A Randomized, Placebo-Controlled, Double-Blind, Parallel-Group, Multicenter, Phase 2a Study of the Efficacy and Safety of Oral AMT-101 in Subjects with Moderate to Severe Ulcerative Colitis (LOMBARD). https://clinicaltrials.gov/study/NCT04583358.

[126] Applied Molecular Transport Applied Molecular Transport Announces Top-Line Phase 2 Results from Lombard Monotherapy Trial of Oral AMT-101 in Patients with Moderate to Severe Ulcerative Colitis. https://www.globenewswire.com/news-release/2022/12/22/2578946/0/en/applied-molecular-transport-announces-top-line-phase-2-results-from-lombard-monotherapy-trial-of-oral-amt-101-in-patients-with-moderate-to-severe-ulcerative-colitis.html.

[127] Applied Molecular Transport Applied Molecular Transport Inc. (Nasdaq: AMTI) (AMT) Today Announced Top-Line Phase 2 Results from the MARKET Combination Trial for AMT-101 in Biologic-Naïve Patients with Moderate-to-Severe UC. https://www.biospace.com/applied-molecular-transport-announces-top-line-phase-2-results-from-market-combination-trial-of-oral-amt-101-in-patients-with-moderate-to-severe-ulcerative-colitis.

[128] Matsumoto H., Haga K., Ohno I., Hiraoka K., Kimura T., Hermann K., Kasahara N., Anton P., McGowan I. (2014). Mucosal gene therapy using a pseudotyped lentivirus vector encoding murine interleukin-10 (mIL-10) suppresses the development and relapse of experimental murine colitis. BMC Gastroenterol..

[129] Zurita-Turk M., del Carmen S., Santos A.C.G., Pereira V.B., Cara D.C., Leclercq S.Y., de LeBlanc A.d., Azevedo V., Chatel J.-M., LeBlanc J.G. (2014). *Lactococcus lactis* carrying the pValac DNA expression vector coding for IL-10 reduces inflammation in a murine model of experimental colitis. BMC Biotechnol..

[130] Martín R., Martín R., Chain F., Chain F., Miquel S., Miquel S., Natividad J.M., Natividad J.M., Sokol H., Sokol H. (2014). Effects in the use of a genetically engineered strain of *Lactococcus lactis* delivering in situ IL-10 as a therapy to treat low-grade colon inflammation. Hum. Vaccin. Immunother..

[131] Steidler L., Neirynck S., Huyghebaert N., Snoeck V., Vermeire A., Goddeeris B., Cox E., Remon J.P., Remaut E. (2003). Biological containment of genetically modified *Lactococcus lactis* for intestinal delivery of human interleukin 10. Nat. Biotechnol..

[132] Mauras A., Chain F., Faucheux A., Ruffié P., Gontier S., Ryffel B., Butel M.-J., Langella P., Bermúdez-Humarán L.G., Waligora-Dupriet A.-J. (2018). A New Bifidobacteria Expression SysTem (BEST) to Produce and Deliver Interleukin-10 in Bifidobacterium bifidum. Front. Microbiol..

[133] Sullivan J.M., Aderhold-Camiré P.A., Brodkin H.R., Nagy-Domonkos C., Dwyer C.J., Hicklin D.J., Lewis Y., Magness L., Nirschl C.J., Steuert Z. (2025). Development of WTX-921, a conditionally activated IL-10 INDUKINETM molecule for the treatment of Inflammatory Bowel Disease 2679. J. Immunol..

[134] Ding Y., Qin L., Kotenko S.V., Pestka S., Bromberg J.S. (2000). A Single Amino Acid Determines the Immunostimulatory Activity of Interleukin 10. J. Exp. Med..

[135] Gorby C., Sotolongo Bellón J., Wilmes S., Warda W., Pohler E., Fyfe P.K., Cozzani A., Ferrand C., Walter M.R., Mitra S. (2020). Engineered IL-10 variants elicit potent immunomodulatory effects at low ligand doses. Sci. Signal..

[136] DiDonato M., Simpson C.T., Vo T., Knuth M., Geierstanger B., Jamontt J., Jones D.H., Fathman J.W., DeLarosa D., Junt T. (2025). A novel interleukin-10 antibody graft to treat inflammatory bowel disease. Structure.

[137] (2025). A Phase 1 First-in-Human Study to Investigate the Safety, Efficacy, Pharmacokinetics, and Pharmacodynamics Activity of FP008 in Subjects with Advanced Solid Tumors. https://clinicaltrials.gov/study/NCT06990698.

[138] Groux H., O’Garra A., Bigler M., Rouleau M., Antonenko S., de Vries J.E., Roncarolo M.G. (1997). A CD4+T-cell subset inhibits antigen-specific T-cell responses and prevents colitis. Nature.

[139] Brun V., Bastian H., Neveu V., Foussat A. (2009). Clinical grade production of IL-10 producing regulatory Tr1 lymphocytes for cell therapy of chronic inflammatory diseases. Int. Immunopharmacol..

[140] (2014). A Phase IIb, Multicentre, Randomised, Double-Blinded, Placebo-Controlled, Multi-Dose and Multi-Injection, Parallel Groups Study to Evaluate the Efficacy and the Safety of Ovasave in Patients with Active Refractory Crohn’s Disease. https://clinicaltrials.gov/study/NCT02327221.

[141] Older E.A., Mitchell M.K., Campbell A., Lian X., Madden M., Wang Y., van de Wal L.E., Zaw T., VanderVeen B.N., Tatum R. (2025). Human gut commensal Alistipes timonensis modulates the host lipidome and delivers anti-inflammatory outer membrane vesicles to suppress colitis in an Il10-deficient mouse model. Gut Microbes.

[142] Golan M.A., Liu W., Shi Y., Chen L., Wang J., Liu T., Li Y.C. (2015). Transgenic Expression of Vitamin D Receptor in Gut Epithelial Cells Ameliorates Spontaneous Colitis Caused by Interleukin-10 Deficiency. Dig. Dis. Sci..

[143] Zegarra Ruiz D.F., Kim D.V., Norwood K., Saldana-Morales F.B., Kim M., Ng C., Callaghan R., Uddin M., Chang L.-C., Longman R.S. (2022). Microbiota manipulation to increase macrophage IL-10 improves colitis and limits colitis-associated colorectal cancer. Gut Microbes.

[144] Kim D.V., Chen A., Ng C., Peres-Tintin L., Park S.-E., Acosta M., Callaghan R., Stanford N., Perez-Rosario M., Matthews D.B. (2025). E. coli-activated cellular circuit restrains intestinal inflammatory microbiota-specific T cells and protects against colitis. Cell Rep..

[145] Xie L.-W., Cai S., Lu H.-Y., Tang F.-L., Zhu R.-Q., Tian Y., Li M. (2024). Microbiota-derived I3A protects the intestine against radiation injury by activating AhR/IL-10/Wnt signaling and enhancing the abundance of probiotics. Gut Microbes.

[146] Yang W., Liu H., Xu L., Yu T., Zhao X., Yao S., Zhao Q., Barnes S., Cohn S.M., Dann S.M. (2022). GPR120 Inhibits Colitis Through Regulation of CD4+ T Cell Interleukin 10 Production. Gastroenterology.

[147] Braat H., Rottiers P., Hommes D.W., Huyghebaert N., Remaut E., Remon J.P., van Deventer S.J.H., Neirynck S., Peppelenbosch M.P., Steidler L. (2006). A Phase I Trial With Transgenic Bacteria Expressing Interleukin-10 in Crohn’s Disease. Clin. Gastroenterol. Hepatol..

[148] Facchin S., Calgaro M., Pandolfo M., De Barba C., Barberio B., Zingone F., Vitulo N., Savarino E.V. (2024). P859 Sodium Butyrate supplementation significantly improved clinical outcomes and quality of life in patients with Crohn’s Disease–results from a randomized placebo-controlled study. J. Crohn’s Colitis.

[149] Sellon R.K., McGhee J.R., Tonkonogy S., Schultz M., Dieleman L.A., Grenther W., Balish E., Rennick D.M., Sartor R.B. (1998). Resident Enteric Bacteria Are Necessary for Development of Spontaneous Colitis and Immune System Activation in Interleukin-10-Deficient Mice. Infect. Immun..

[150] Atreya I., Neurath M.F. (2024). Linking IL-10 signaling with lipid metabolic programs in macrophages: Dysregulated ceramide homeostasis drives colitis. Signal Transduct. Target. Ther..

[151] Chiappa N.F., Lal N., Botchwey E.A. (2025). Resolving versus non-resolving sphingolipid dynamics during macrophage activation: A time-resolved metabolic analysis. J. Lipid Res..

[152] Veenbergen S., Li P., Raatgeep H.C., Lindenbergh-Kortleve D.J., Simons-Oosterhuis Y., Farrel A., Costes L.M.M., Joosse M.E., van Berkel L.A., de Ruiter L.F. (2019). IL-10 signaling in dendritic cells controls IL-1β-mediated IFNγ secretion by human CD4+ T cells: Relevance to inflammatory bowel disease. Mucosal Immunol..

[153] Narumi Y., Isomoto H., Shiota M., Sato K., Kondo S., Machida H., Yanagihara K., Mizuta Y., Kohno S., Tsukamoto K. (2009). Polymorphisms of PTPN11 coding SHP-2 as biomarkers for ulcerative colitis susceptibility in the Japanese population. J. Clin. Immunol..

[154] Lai W., Zhu X., Yi Y., Zhu Y., Hu S., Mao R., Chen M., Feng R. (2026). P0030 Fecal Lipidomics Identifies Lactosylceramide as a Disease-Associated Glycosphingolipid with Diagnostic and Mechanistic Relevance in Crohn’s Disease. J. Crohn’s Colitis.

[155] Li Q., Li J., Liu S., Zhang Y., Liu J., Wan X., Liang G. (2025). Molecular Subtypes and Biomarkers of Ulcerative Colitis Revealed by Sphingolipid Metabolism-Related Genes: Insights from Machine Learning and Molecular Dynamics. Curr. Issues Mol. Biol..

[156] Bozic J., Santic R., Zivkovic P.M., Kumric M. (2026). Diet, Gut Microbiome, and Microbial Metabolites in Inflammatory Bowel Disease: From Functional Dysbiosis to Precision Nutrition. Int. J. Mol. Sci..

[157] Moraga I., Wernig G., Wilmes S., Gryshkova V., Richter C.P., Hong W.-J., Sinha R., Guo F., Fabionar H., Wehrman T.S. (2015). Tuning Cytokine Receptor Signaling by Re-orienting Dimer Geometry with Surrogate Ligands. Cell.

[158] Wang Y., Sun S.N., Liu Q., Yu Y.Y., Guo J., Wang K., Xing B.C., Zheng Q.F., Campa M.J., Patz E.F. (2016). Autocrine Complement Inhibits IL10-Dependent T-cell-Mediated Antitumor Immunity to Promote Tumor Progression. Cancer Discov..

[159] Garrido-Trigo A., Corraliza A.M., Veny M., Dotti I., Melón-Ardanaz E., Rill A., Crowell H.L., Corbí Á., Gudiño V., Esteller M. (2023). Macrophage and neutrophil heterogeneity at single-cell spatial resolution in human inflammatory bowel disease. Nat. Commun..

